# Cellular co-infections of West Nile virus and Usutu virus influence virus growth kinetics

**DOI:** 10.1186/s12985-023-02206-9

**Published:** 2023-10-13

**Authors:** Christin Körsten, Hannah Reemtsma, Ute Ziegler, Susanne Fischer, Birke A. Tews, Martin H. Groschup, Cornelia Silaghi, Ana Vasic, Cora M. Holicki

**Affiliations:** 1 Federal Research Institute for Animal Health, Institute of Infectology, Friedrich-Loeffler-Institut, 17493 Greifswald-Insel Riems, Germany; 2 Federal Research Institute for Animal Health, Institute of Novel and Emerging Infectious Diseases, Friedrich-Loeffler-Institut, 17493 Greifswald-Insel Riems, Germany; 3 Scientific Institute of Veterinary Medicine of Serbia, Belgrade, Serbia

**Keywords:** *Flaviviruses*, *In-vitro*, West Nile virus, Usutu virus, Co-infection, Viral interference

## Abstract

The mosquito-borne flaviviruses West Nile virus (WNV) and Usutu virus (USUV) pose a significant threat to the health of humans and animals. Both viruses co-circulate in numerous European countries including Germany. Due to their overlapping host and vector ranges, there is a high risk of co-infections. However, it is largely unknown if WNV and USUV interact and how this might influence their epidemiology. Therefore, *in-vitro* infection experiments in mammalian (Vero B4), goose (GN-R) and mosquito cell lines (C6/36, CT) were performed to investigate potential effects of co-infections in vectors and vertebrate hosts. The growth kinetics of German and other European WNV and USUV strains were determined and compared. Subsequently, simultaneous co-infections were performed with selected WNV and USUV strains. The results show that the growth of USUV was suppressed by WNV in all cell lines. This effect was independent of the virus lineage but depended on the set WNV titre. The replication of WNV also decreased in co-infection scenarios on vertebrate cells. Overall, co-infections might lead to a decreased growth of USUV in mosquitoes and of both viruses in vertebrate hosts. These interactions can strongly affect the epidemiology of USUV and WNV in areas where they co-circulate.

**Supplementary Information:**

The online version contains supplementary material available at 10.1186/s12985-023-02206-9.

## Introduction

West Nile virus (WNV) and Usutu virus (USUV; genus *Flavivirus*, family *Flaviviridae*) are two closely related zoonotic mosquito-borne viruses. Both circulate in an enzootic cycle between mosquitoes as biological vectors and birds as primary vertebrate hosts, but can also be transmitted to other mammalian species [1]. Infections with WNV in horses and humans can cause various clinical pictures, including severe neurological diseases [2]. Symptomatic USUV infections with neurological disorders have been observed in humans only in individual cases, however, in recent years, the number of USUV-infected human cases in Europe has steadily increased [3].

WNV lineage 1 strains have been circulating in Europe for several decades [4]. In 2004, WNV lineage 2 was detected for the first time in Hungary [5] and has since then continued to spread throughout Europe [6]. In Germany, WNV lineage 2 has been circulating since 2018, causing infections in birds, horses and humans every year [7–10]. Similarly to WNV, USUV was first detected in Europe in Austria in 2001 [11], but retrospective analysis of historical bird tissues had shown that the virus was already present in Italy in 1996 [12]. Since the first detection of USUV in Germany in 2010 [13], the virus spread nationwide within a few years, causing significant numbers of bird deaths, especially in blackbirds (*Turdus merula*) [14–16]. Currently several USUV lineages are circulating in Germany, with USUV lineages Europe 3 and Africa 3 predominating in all federal states [9, 14].

The distribution areas of WNV and USUV are increasingly overlapping in central European countries such as in Germany [9] as well as in several other countries [1, 17]. In addition to their geographical co-circulation, WNV and USUV are also epidemiologically closely-related, sharing the same vertebrate hosts and mosquito vectors [1, 17]. The risk of co-infections with both viruses therefore exists, and indeed co-infections in birds [18, 19] and humans [10, 20] have already been reported.

*In-vitro* studies are a fundamental first step in investigating viral co-infections and their effects on virus replication. A few previously conducted *in-vitro* co-infection studies examined combinations of WNV with other flaviviruses [21, 22], but to date only one study investigated co-infections with WNV and USUV [23]. In this study, it was shown that the replication of USUV Africa 3 was inhibited by WNV lineage 2 in mammalian, avian and mosquito cells [23]. However, due to the co-circulation of two WNV lineages and several USUV lineages in Europe, a combination of just one lineage per virus quickly reaches its limitations in experimentally reflecting the actual situation in Europe. Further studies were necessary to investigate and understand the interactions between WNV and USUV. The aim of this study was therefore to examine growth kinetics of a range of WNV and USUV lineages and isolates and to analyse co-infections of selected viral isolates in mammalian, avian and mosquito cell lines.

## Materials and methods

### Cells and viruses

For the mono- and co-infection kinetics of WNV and USUV, the well-established mammalian (Vero B4) and mosquito (C6/36) cell lines were used (Additional file 1: TableS1). Furthermore, specific cells derived from a potential host (domestic geese (*Anser anser f. domestica*)) for both WNV and USUV [24–26] and from a potential vector species (western encephalitis mosquito (*Cx. tarsalis*)) [27] were utilized (GN-R and CT, respectively). Growth kinetics were performed with various German USUV (Africa 3 and Europe 3) and WNV (lineage 2) strains. Furthermore, an Austrian WNV isolate (lineage 2) and an Italian WNV isolate (lineage 1) were also assessed (Additional file 1: Table S2).

### Kinetics of viral secretion

**Fig. 1:**
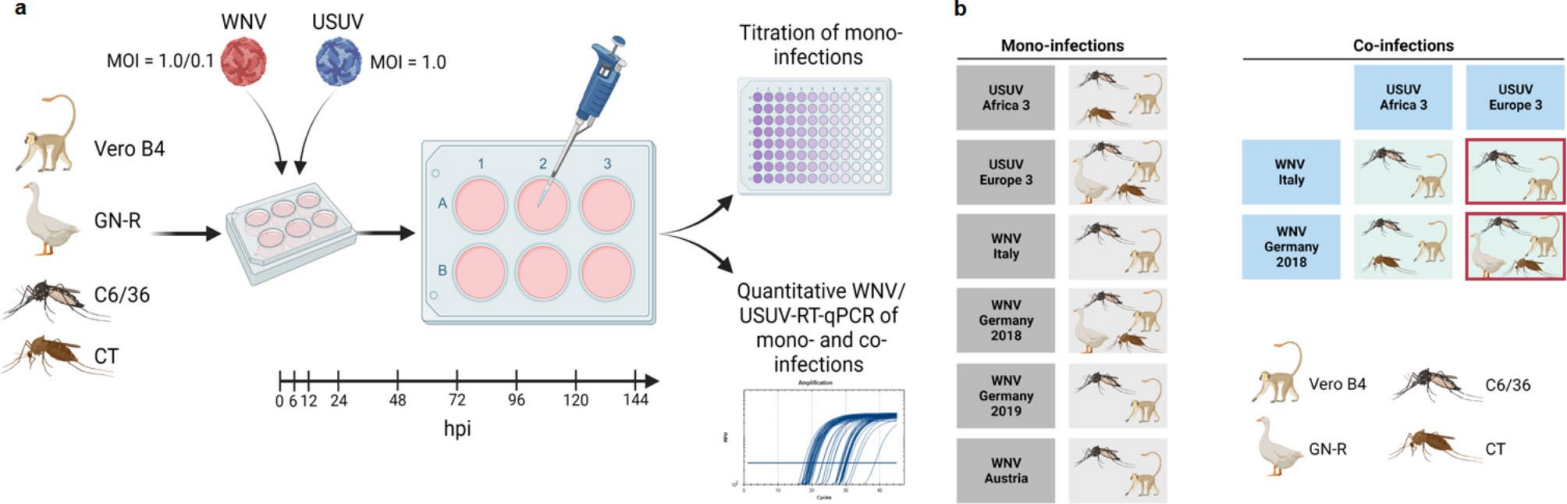
Workflow of *in-vitro* infections and performed mono- and co-infections in this study. (**a**) Workflow of mono- and co-infections (simultaneous) using Vero B4, GN-R, C6/36 and CT cells. (**b**) Display of the performed infection experiments with focus on virus combinations (WNV versus USUV). Boxes with a grey background display the mono-infections, boxes with a blue background display the co-infections and boxes framed in red show where two different MOIs for WNV were used (1 or 0.1). Created with Biorender.com

Cells were seeded on 6-well cell culture plates (Corning® Costar® TC-Treated Multiple Well Plates CLS3516; Sigma-Aldrich Chemie GmbH, Taufkirchen, Germany) 24 h prior to infection at the set concentrations (Additional file 1: TableS1). For each of the three biological replicates the following procedure was repeated. On the day of infection, the cells of one well were used to determine the cell count. Accordingly, the remaining wells, except for one control well, were washed with phosphate-buffered-saline (PBS) and infected with virus at the desired multiplicity of infection (MOI). For the mono-infections and simultaneous co-infections, a MOI of 1 was used. After 1 h of incubation, each well was washed with PBS three times and refilled with medium (listed in Additional file 1: TableS1) supplemented with 2% fetal calf serum (and 1% chicken serum for the GN-R cells) and antibiotics (penicillin/streptomycin; Merck, St. Louis, MO, USA). At 0, 6, 12, 24, 48, 72, 96, 120 and 144 h post infection (hpi) the medium of the corresponding well was transferred into 2 × 2 mL screw-cap-tubes. Afterwards they were centrifuged at 2,500 rpm, for 10 min at 12 °C (5430R centrifuge; Eppendorf, Hamburg, Germany) and the supernatant was aliquoted into 4 × 500 µL in 2 mL cryo tubes and 2 × 140 µL in 560 µL AVL-Buffer (Qiagen, Hilden, Germany) and frozen at -80 °C. The performed mono- and simultaneous co-infections and the exact workflow are depicted in Fig. 1. An additional co-infection with WNV Germany 2018 or WNV Italy 2009 and USUV Europe 3 was also completed with a lower WNV titre (MOI of only 0.1) in combination with an unaltered USUV titre (MOI of 1).

### Sample processing

The supernatants from mono-infections were analysed via virus titration with an endpoint dilution assay on Vero B4 cells. After seven days of incubation, the cells were formalin-fixed, stained with crystal violet and the virus titres were calculated with the Spearman-Kaerber method. The samples in AVL were heat-inactivated for 10 min at 70 °C using Eppendorf ThermoMixer compact (Eppendorf, Hamburg, Germany). Viral RNA was then extracted using the NucleoMag Vet Kit (Macherey-Nagel, Düren, Germany) and BioSprint96 (Qiagen, Hilden, Germany) according to the manufacturer´s instructions. The RNA extracts were either examined with the specific USUV RT-qPCR [13] or the specific WNV RT-qPCR for the simultaneous detection of WNV lineages 1 and 2 [28], or with both. For the quantification of viral RNA copies in each sample, a calibration curve of synthetic WNV and USUV RNA was run in parallel using 6-fold serial dilutions (i.e., relative standard) [28, 29]. Additionally, per tested virus an aliquot of the according stock was diluted, extracted and used to estimate the 50% tissue culture infectious dose per mL (TCID_50_/mL) of the virus stock (i.e., absolute standard) (Additional file 1: Tables S3, S4).

### Statistics

Data visualization, analysis and statistics were conducted in R (v3.6.2, x64)/R Studio (Version 1.4.1103) [30, 31]. Data from RT-qPCR (TCID_50_; derived from the relative and absolute standard curves) or titration analyses (TCID_50_) were log transformed and checked for homogeneity of variance across groups by the Levene Test. Following a generalized linear model (RT-qPCR versus titration comparisons) or a multifactorial analysis of variance (ANOVA) (co-infection analyses only using the data from RT-qPCR) with gamma distribution was applied [32]. Pairwise analyses were attached by least square means analyses for multiple comparisons under the lsmean package with tukey adjustment [33]. Results are interpreted as significant if: p-value ≤ 0.05.

## Results

### Growth kinetics of WNV and USUV strains on different cell lines

**Fig. 2:**
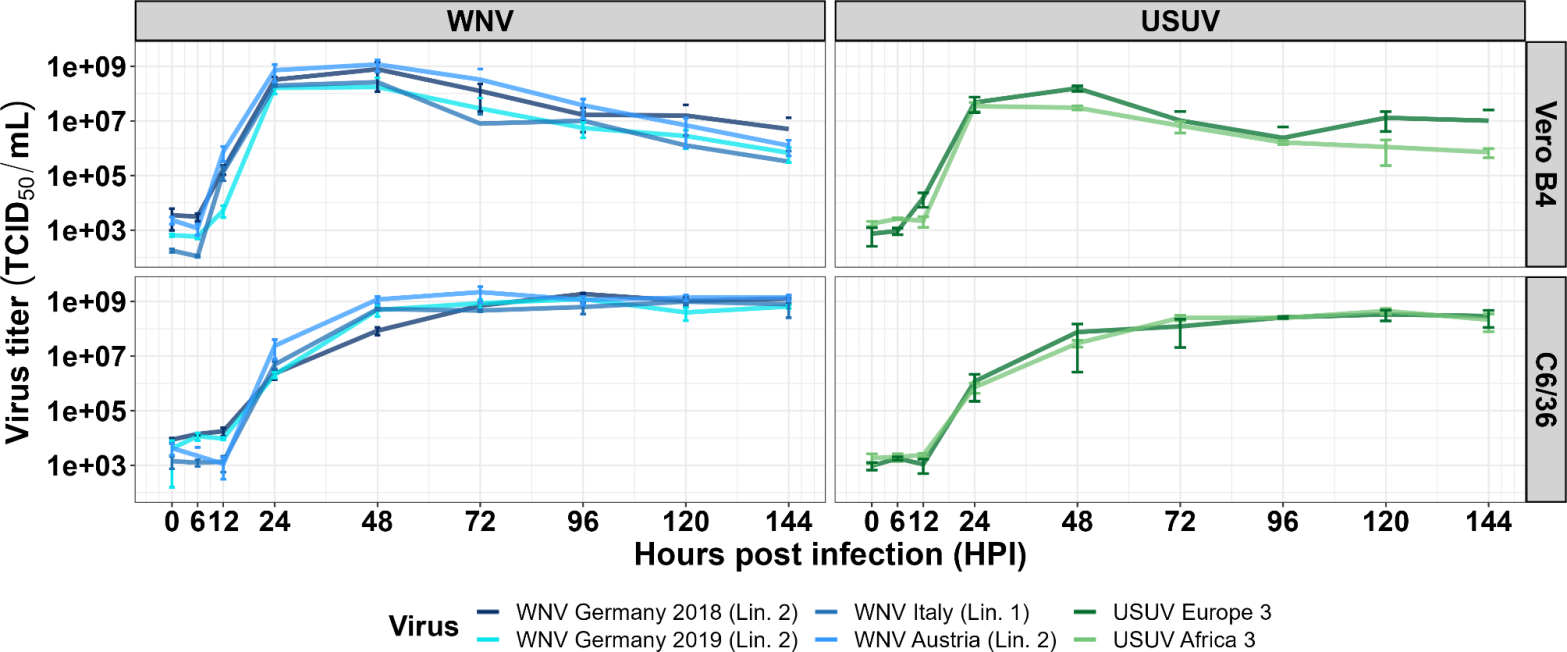
Growth kinetics of West Nile viruses (WNV) and Usutu viruses (USUV). All mono-infections were performed with a multiplicity of infection (MOI) of 1. The solid lines are drawn through the mean values of the three biological replicates for all tested time points measured by virus titration. The error bars represent the standard deviation (± SD). Incomplete error bars occur when y-min of the error bars is negative and therefore not displayed in the logarithmic y-scale

Growth kinetics (mono-infections) were performed with all USUV and WNV strains on Vero B4 and C6/36 (Fig. 2; Additional file 1: Table S2). Furthermore, mono-infections with a German WNV strain (lineage 2) and a German USUV strain (Europe 3) were also carried out on GN-R and CT cells (Fig. 1). Morphological changes of the individual cell lines during the experiments were comparable for all of the tested WNV and USUV strains. The strongest cytopathic effects (CPE) were observed in the GN-R cell line, followed by the Vero B4 cell line (Additional file 1: Fig. S1). In contrast to both vertebrate cell lines, only very slight indications for a CPE (increased cell agglomerates) were observed in the two mosquito cell lines (C6/36 and CT) (Additional file 1: Fig. S1).

All tested WNV lineages replicated faster to higher viral titres in mammalian (Vero B4) and mosquito cell lines (C6/36) than the two USUV strains (Fig. 2). As shown in Fig. 2, the course of the viral replication curves of each WNV and USUV isolate is similar. However, there were individual statistically significant differences between the WNV as well as USUV strains with regard to time points, cell lines and genetic lineages (Additional file xindividual statistically significant differences between: Tables S5, S6, S7>). For example, there was an increased rate of virus replication up to 24 hpi in Vero B4 cells, when compared to C6/36 cells. However, after reaching their maximum values the virus titres steadily declined on the Vero B4, in accordance with observed cell death (Additional file 1: Fig. S1). Similar results were found for the growth kinetics on GN-R (Additional file 1: Fig. S2).

### Titration versus RT-qPCR

The agreement of the titres determined by titration and RT-qPCR (calculated via the absolute and relative standard curves; Additional file 1: Tables S3, S4>) varied between the virus strains, cell lines and sampling time points. The optimal accordance between the titration and RT-qPCR results occurred around peak viral titre, at 24, 48 and 72 hpi (Additional file 1: Tables S8, S9). On the basis of this data, all further statistical evaluations focused on these three time points. For the subsequent analyses between mono- and co-infections only data from the RT-qPCRs were used.

### The effect of WNV and USUV co-infections on viral replication under two different MOI-conditions

**Fig. 3:**
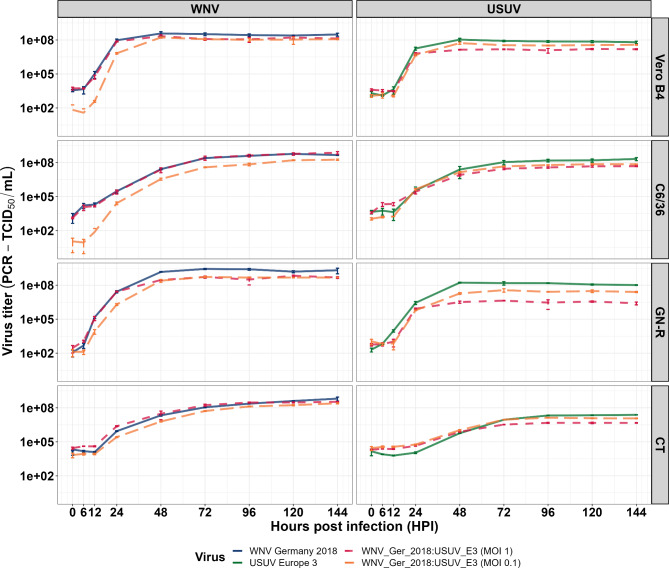
Virus secretion in mono- and co-infections of West Nile virus (WNV) and Usutu virus (USUV). WNV lineage 2 isolated in Germany in 2018 and USUV Europe 3 isolated in Germany in 2011 were used for co-infections in vertebrate (Vero B4 GN-R) as well mosquito cell lines (C6/36 and CT). Co-infections were performed with a multiplicity of infection (MOI) of 1 for USUV and either 1 or 0.1 for WNV. The solid and dashed lines are drawn through the mean values of the three biological replicates for all tested time points measured by RT-qPCR based on a relative and absolute standard curve running in parallel. The error bars represent the standard deviation (± SD). Incomplete error bars occur when y-min of the error bars is negative and therefore not displayed in the logarithmic y-scale

When co-infected on the vertebrate cell lines (Vero B4, GN-R) the WNV and USUV viral titres were lower compared to those of the single infection (Fig. 3). Interestingly, however, when co-infected the WNV viral titres converged from 48 hpi onwards irrespective of whether the same or a lower MOI was used for WNV than for USUV (no statistically significant differences; Additional file 1: Table S10). In the insect cell lines (C6/36, CT), USUV viral titres were reduced in co-infections with WNV, while WNV titres only depended on the WNV MOI but not on the simultaneous occurrence of USUV (Fig. 3). Thus, the used MOI for WNV had an impact on the subsequent USUV titre in the co-infection experiments primarily in the vertebrate cell lines and to some extent in the CT cells (p < 0.0001; Additional file 1: Table S11). The USUV viral titres were significantly higher when a lower MOI for WNV was used (0.1 rather than 1) (Additional file 1: Tables S10, S11, S12, S13, S14). This effect was most pronounced in the avian cell line (GN-R; Fig. 3), where there was a statistically significant difference between the virus titres of mono- and co-infected as well as between a higher and lower WNV MOI (except for 24 hpi; p < 0.0001; Additional file 1: Table S15).

### Comparison of mono- and co-infections with various virus combinations

**Fig. 4:**
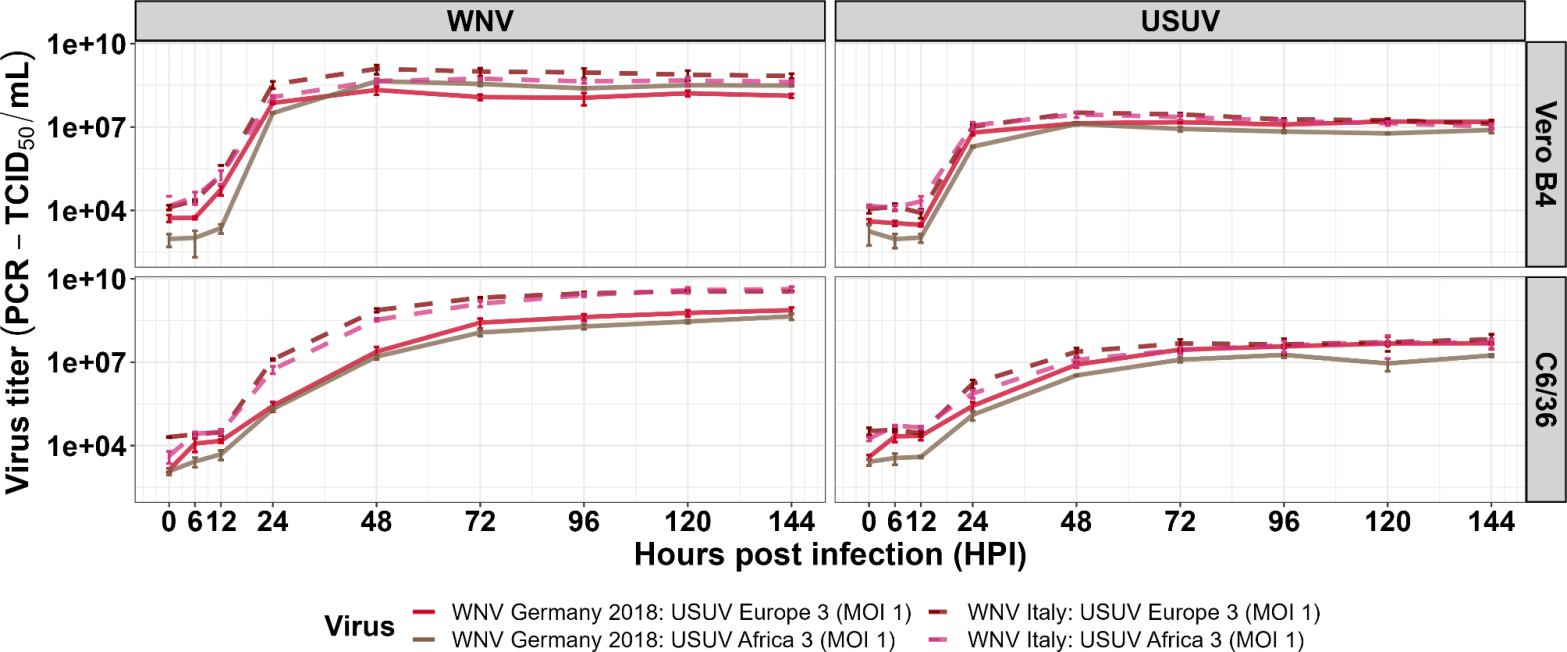
Virus secretion in co-infections of different West Nile virus (WNV) and Usutu virus (USUV) lineages. Co-infections were performed with WNV lineage 1 (Italy, 2009) and 2 (Germany, 2018) and with USUV Europe 3 (Germany, 2011) and Africa 3 (Germany, 2016) in vertebrate (Vero B4) as well as mosquito cell lines (C6/36). Co-infections were performed with a multiplicity of infection (MOI) of 1 for both viruses. The solid and dashed lines are drawn through the mean values of the three biological replicates for all tested time points measured by RT-qPCR based on a relative and absolute standard curve running in parallel. The error bars represent the standard deviation (± SD). Incomplete error bars occur when y-min of the error bars is negative and therefore not displayed in the logarithmic y-scale

When comparing the co-infections of WNV lineage 2 from Germany (2018) or WNV linage 1 from Italy (2009) with both USUV lineages, respectively, results were similar although less prominent (Fig. 3; Additional file 1: Fig.S3, S4, S5, Tables S16, S17, S18, S19, S20, in part only performed on Vero B4 and C6/36 cells). For example, WNV lineage 1 from Italy had a similar effect to WNV lineage 2 from Germany (2018) on USUV Europe 3 replication when co-infected on Vero B4 and C6/36 cells (Fig. 3; Additional file 1: Fig.S3). For the two different WNV lineages, the course of viral replication was similar independent of the USUV strain used (Fig. 4). However, in the C6/36 the increase in viral genome copy numbers was more rapid for the co-infection of WNV lineage 1 Italy with USUV than for WNV lineage 2 Germany 2018. This can be attributed to the more efficient replication of WNV lineage 1 from Italy independent of the presence of USUV, as already observed in the mono-infections (Fig. 2).

## Discussion

With the introduction of WNV into Germany, there is a need to understand the role that co-infections with WNV and USUV might play in the enzootic transmission cycle. The focus of this study was therefore to investigate the viral replication as well as potential interactions in co-infections of German and other European WNV and USUV strains.

All WNV and USUV isolates demonstrated a rapid viral growth followed by a steady decline of the titres due to a strong CPE in both vertebrate cell lines. Maximum titres were higher in the avian GN-R cells than in the mammalian Vero B4 cells, similar to results from previous studies [34]. This is consistent with the more efficient viral replication of WNV and USUV in avian species compared to mammals [35], although geese are not the primary hosts for these viruses [36]. In contrast, there was a slower but steady viral growth in both mosquito cell lines, with limited CPE (low levels to none detected), which matches the life-long viral infectivity of mosquito vectors [37]. The observed growth of WNV and USUV on these cell lines are in accordance to the potential vector competence of *Aedes albopictus* for WNV and USUV [38, 39] and *Culex tarsalis* for WNV [40]. Overall, the observed differences between insect and vertebrate cells were already described in previous *in-vitro* experiments [23, 41]. Decisive factors for the different viral replication kinetics might be inherent differences in viral replication in mammalian versus insect cells as well as the used incubation temperatures [42, 43]. *In-vitro* attenuations of the virus isolates to certain cell lines must also not be disregarded.

When comparing the viral growth kinetics within one virus species, USUV Europe 3 demonstrated slower viral growth than USUV Africa 3 on CT cells but not on C6/36 cells. A possible explanation might be the presence of a functional RNA interference pathway, which is the main antiviral pathway in mosquitoes [44], and proved to be sufficient in CT but not C6/36 cells [37, 45]. Apart from that, there were no differences in the viral replication kinetics. This is in accordance with *in-vivo* observations in geese where two different WNV strains, whose isolates were also used in the current study, caused comparable pathology [25, 26]. In contrast, another *in-vitro* study reported differences in viral replication of USUV strains in cell culture as well as virulence in mice [46]. Overall, all WNV strains replicated faster and to higher maximum titres than the USUV strains on all tested cell lines. This result has also been reported from other cell experiments [23, 47] and possibly explains the higher number of WNV deceased birds [9] as well as the higher disease severity of WNV in humans [10].

Other *in-vitro* co-infection studies with flaviviruses mostly reported a competition between both viruses, resulting in the inhibition of at least one virus [23, 48, 49]. Similarly, a competition between WNV and USUV could be observed in this study, with a decreased replication of USUV in all cell lines. The suppression of USUV was most evident in the avian cells. The faster replication of WNV observed in the mono-infections appears to have caused a competitive advantage of WNV over USUV. Due to their genetic and phylogenetic relationship, WNV and USUV likely use the same cell receptors and/or components for their replication [37], resulting in a competition for these resources in both host and vector cells. Similarly, closely-related viruses can activate identical cellular defences, in turn cross-protecting cells against an additional infection [50]. This is also supported by the fact that the suppression of USUV was dependent on WNV MOI, where a lower concentration of WNV particles might enable USUV to initially infect more cells, resulting in a higher maximum titre.

The viral interference appeared not to be strain or lineage dependent as similar results were found for combinations with other virus isolates. This was not surprising as almost all strains had similar viral kinetics. It must be noted that, even though marked differences in virulence were not observed between the virus strains used in this study, it is not uncommon for WNV to show variances in its efficiency to replicate and become neuroinvasive, as shown for Australian strains in cells and an established mouse model [51]. Interestingly, however, even the different growth kinetics of both USUV lineages on CT cells did not have an impact on the outcome. Therefore, competition for resources seems to be more likely than a potential impact of RNA interference. In the vertebrate cells (Vero B4 and GN-R), the growth of WNV was also reduced when the cells were co-infected with USUV. However, it remains unclear if this WNV-reduction was caused by USUV or by the general loss of viable vertebrate cells over time. Since WNV growth did not appear to be affected in the co-infected insect cell lines (CT and C6/36) the latter explanation is more likely.

Overall, WNV appears to have an advantage over USUV, possibly due to the observed different replication kinetics in host and vector cells. This is in accordance to *in-vivo* findings in birds and mosquitoes. Birds that were co-infected with both viruses had higher viral loads of WNV than USUV [18], and USUV infection was reduced in *Cx. pipiens* biotype *pipiens* that were simultaneously infected with WNV [23, 52]. Taken together with the results of this study, WNV proves to be a virus with a high viral fitness, possessing the ability to replicate rapidly and efficiently in a broad range of host and vector cells. It can outcompete closely related viruses such as USUV. This might also be one of the reasons for its unprecedented worldwide distribution to date. However, there are still some unanswered questions. Although the viral interference between WNV and USUV was confirmed in all mosquito cell lines, the suppression of USUV in mosquitoes *in-vivo* could not be confirmed for every mosquito species [52]. Similarly, co-infections in mammalian and avian species might lead to unpredictable outcomes. The exact cellular mechanisms underlying the interactions between WNV and USUV remain unexplained and should be targeted by future investigations.

### Electronic supplementary material

Below is the link to the electronic supplementary material.

**Supplementary material: additional file 1**: Fig.S1. Cytopathic effects of WNV lineage 2 (Germany, 2018) in mammalian, avian and mosquito cell lines. Fig. S2. Growth kinetics of West Nile virus (WNV) and Usutu virus (USUV) on various cell lines. Fig. S3. Virus secretion in mono- and co-infections of WNV lineage 1 and USUV Europe 3. Fig. S4. Virus secretion in mono- and co-infections of WNV lineage 2 and USUV Africa 3. Fig. S5. Virus secretion in mono- and co-infections of WNV lineage 1 and USUV Africa 3. Table S1. Description of the cell lines and media used in the study. Table S2. Description of the virus stocks used in the study. Table S3. Table comparing standard curves of the synthetic and naïve standards for Usutu virus (USUV). Table S4. Table comparing standard curves of the synthetic and naïve standards for West Nile virus (WNV). Table S5. Dependency of viral replication on the virus strain, time point and the infected cell line. Table S6. Dependence of WNV replication on constellation of the respective WNV mono-infections and the time points. Table S7. Dependency of USUV viral replication on the cells at the different time points. Table S8. Comparison of virus titres examined by virus titration or RT-qPCR on Vero B4 cells. Table S9. Comparison of virus titres examined by virus titration or RT-qPCR on C6/36 cells. Table S10. Comparison of virus combinations at different time points for Vero B4 cells. Table S11. Dependency of viral replication on the multiplicity of infection and time point. Table S12. Dependency of USUV viral replication on the virus constellation and time points in CT cells. Table S13. Dependency of WNV viral replication on the virus constellation and time points in CT cells. Table S14. Dependence of virus combinations and different time points for C6/36 cells. Table S15. Dependency of viral replication on the virus constellation and the time points in GN-R. Table S16. Dependency of viral replication on virus constellation and time point in Vero B4 and C6/36. Table S17. Dependency of WNV viral replication on the virus constellation and time points in Vero B4. Table S18. Dependency of USUV viral replication on the virus constellation and time points in Vero B4. Table S19. Dependency of WNV viral replication on the virus constellation and time points in C6/36. Table S20. Dependency of USUV viral replication on the virus constellation and time points in C6/36

## Data Availability

All data generated or analysed during this study are included in this published article and its supplementary information files.

## List of abbreviations

CPE: cytopathic effect
hpi: hours post infection
MOI: multiplicity of infection
PBS: phosphate-buffered saline
RT-qPCR: quantitative reverse transcriptase polymerase chain reaction
TCID50/mL: 50% tissue culture infectious dose
USUV: Usutu virus
WNV: West Nile virus

## References

[1] Nikolay B (2015). A review of West Nile and Usutu virus co-circulation in Europe: how much do transmission cycles overlap?. Trans R Soc Trop Med Hyg.

[2] Byas AD, Ebel GD (2020). Comparative Pathology of West Nile Virus in humans and non-human animals. Pathogens.

[3] Cadar D, Simonin Y (2023). Human Usutu Virus Infections in Europe: a New Risk on Horizon?. Viruses.

[4] Hubálek Z, Halouzka J (1999). West Nile fever–a reemerging mosquito-borne viral disease in Europe. Emerg Infect Dis.

[5] Bakonyi T, Ivanics E, Erdélyi K, Ursu K, Ferenczi E, Weissenböck H, Nowotny N (2006). Lineage 1 and 2 strains of encephalitic West Nile virus, central Europe. Emerg Infect Dis.

[6] European Centre for Disease Prevention and Control. West Nile virus infection. Annual Epidemiological Report for 2019. Stockholm; 2021.

[7] Ziegler U, Lühken R, Keller M, Cadar D, van der Grinten E, Michel F (2019). West Nile virus epizootic in Germany, 2018. Antiviral Res.

[8] Ziegler U, Santos PD, Groschup MH, Hattendorf C, Eiden M, Höper D (2020). West Nile Virus Epidemic in Germany triggered by epizootic emergence, 2019. Viruses.

[9] Ziegler U, Bergmann F, Fischer D, Müller K, Holicki CM, Sadeghi B (2022). Spread of West Nile Virus and Usutu Virus in the german Bird Population, 2019–2020. Microorganisms.

[10] Frank C, Schmidt-Chanasit J, Ziegler U, Lachmann R, Preußel K, Offergeld R (2022). West Nile Virus in Germany: an emerging infection and its relevance for Transfusion Safety. Transfus Med Hemother.

[11] Weissenböck H, Kolodziejek J, Url A, Lussy H, Rebel-Bauder B, Nowotny N (2002). Emergence of Usutu virus, an african mosquito-borne flavivirus of the japanese encephalitis virus group, central Europe. Emerg Infect Dis.

[12] Weissenböck H, Bakonyi T, Rossi G, Mani P, Nowotny N (2013). Usutu virus, Italy, 1996. Emerg Infect Dis.

[13] Jöst H, Bialonski A, Maus D, Sambri V, Eiden M, Groschup MH (2011). Isolation of usutu virus in Germany. Am J Trop Med Hyg.

[14] Michel F, Sieg M, Fischer D, Keller M, Eiden M, Reuschel M (2019). Evidence for West Nile Virus and Usutu Virus Infections in Wild and Resident birds in Germany, 2017 and 2018. Viruses.

[15] Sieg M, Schmidt V, Ziegler U, Keller M, Höper D, Heenemann K (2017). Outbreak and cocirculation of three different Usutu virus strains in Eastern Germany. Vector Borne Zoonotic Dis.

[16] Becker N, Jöst H, Ziegler U, Eiden M, Höper D, Emmerich P (2012). Epizootic emergence of Usutu virus in wild and captive birds in Germany. PLoS ONE.

[17] Zannoli S, Sambri V (2019). West Nile Virus and Usutu Virus Co-Circulation in Europe: Epidemiology and Implications. Microorganisms.

[18] Santos PD, Michel F, Wylezich C, Höper D, Keller M, Holicki CM, et al. Co-infections: simultaneous detections of West Nile virus and Usutu virus in birds from Germany. Transbound Emerg Dis. 2021;1–17. 10.1111/tbed.14050. 10.1111/tbed.14050 33655706

[19] Lauriano A, Rossi A, Galletti G, Casadei G, Santi A, Rubini S (2021). West Nile and Usutu Viruses’ Surveillance in birds of the Province of Ferrara, Italy, from 2015 to 2019. Viruses.

[20] Aberle SW, Kolodziejek J, Jungbauer C, Stiasny K, Aberle JH, Zoufaly A (2018). Increase in human West Nile and Usutu virus infections, Austria, 2018. Euro Surveill.

[21] Goenaga S, Goenaga J, Boaglio ER, Enria DA, Del Levis SC (2020). Superinfection exclusion studies using West Nile virus and Culex flavivirus strains from Argentina. Mem Inst Oswaldo Cruz.

[22] Colmant AMG, Hall-Mendelin S, Ritchie SA, Bielefeldt-Ohmann H, Harrison JJ, Newton ND (2018). The recently identified flavivirus Bamaga virus is transmitted horizontally by Culex mosquitoes and interferes with West Nile virus replication in vitro and transmission in vivo. PLoS Negl Trop Dis.

[23] Wang H, Abbo SR, Visser TM, Westenberg M, Geertsema C, Fros JJ (2020). Competition between Usutu virus and West Nile virus during simultaneous and sequential infection of Culex pipiens mosquitoes. Emerg Microbes Infect.

[24] Swayne DE, Beck JR, Smith CS, Shieh WJ, Zaki SR (2001). Fatal encephalitis and myocarditis in young domestic geese (Anser anser domesticus) caused by West Nile virus. Emerg Infect Dis.

[25] Holicki CM, Michel F, Vasić A, Fast C, Eiden M, Răileanu C (2020). Pathogenicity of West Nile Virus lineage 1 to german poultry. Vaccines (Basel).

[26] Reemtsma H, Holicki CM, Fast C, Bergmann F, Eiden M, Groschup MH, Ziegler U (2022). Pathogenesis of West Nile Virus lineage 2 in domestic geese after experimental infection. Viruses.

[27] Chao J, Ball GH. Comparison of amino acid utilization by cell lines of Culex tarsalis and of Culex pipiens. Invertebrate Tissue Culture. 1976;263–6. 10.1016/B978-0-12-429740-1.50028-X.

[28] Eiden M, Vina-Rodriguez A, Hoffmann B, Ziegler U, Groschup MH (2010). Two new real-time quantitative reverse transcription polymerase chain reaction assays with unique target sites for the specific and sensitive detection of lineages 1 and 2 West Nile virus strains. J Vet Diagn Invest.

[29] Pinho Dos Reis V, Keller M, Schmidt K, Ulrich RG, Groschup MH (2021). αVβ3 integrin expression is essential for replication of Mosquito and Tick-Borne Flaviviruses in Murine Fibroblast cells. Viruses.

[30] RStudio Team. RStudio: Integrated Development for R. 2019. http://www.rstudio.com/.

[31] R Core Team. R: A language and environment for statistical computing. 2019. https://www.R-project.org/.

[32] Fox J, Weisberg S (2018). An R companion to applied regression.

[33] Lenth RV (2016). Least-Squares Means: the R Package lsmeans. J Stat Soft.

[34] Bakonyi T, Lussy H, Weissenböck H, Hornyák A, Nowotny N (2005). In vitro host-cell susceptibility to Usutu virus. Emerg Infect Dis.

[35] Hayes EB, Komar N, Nasci RS, Montgomery SP, O’Leary DR, Campbell GL (2005). Epidemiology and transmission dynamics of West Nile virus disease. Emerg Infect Dis.

[36] Chvala S, Bakonyi T, Hackl R, Hess M, Nowotny N, Weissenböck H (2006). Limited pathogenicity of usutu virus for the domestic goose (Anser anser f. domestica) following experimental inoculation. J Vet Med B Infect Dis Vet Public Health.

[37] Salas-Benito JS, de Nova-Ocampo M (2015). Viral interference and persistence in Mosquito-Borne Flaviviruses. J Immunol Res.

[38] Holicki CM, Ziegler U, Răileanu C, Kampen H, Werner D, Schulz J (2020). West Nile virus lineage 2 vector competence of indigenous Culex and Aedes mosquitoes from Germany at temperate climate conditions. Viruses.

[39] Martinet J-P, Bohers C, Vazeille M, Ferté H, Mousson L, Mathieu B et al. Assessing vector competence of mosquitoes from northeastern France to West Nile virus and Usutu virus 2023. 10.1101/2023.02.07.527438. 10.1371/journal.pntd.0011144 PMC10270612 37276229

[40] Reisen WK, Fang Y, Martinez VM (2006). Effects of temperature on the transmission of West Nile Virus by Culex tarsalis (Diptera: Culicidae). J Med Entomol.

[41] Bates TA, Chuong C, Hawks SA, Rai P, Duggal NK, Weger-Lucarelli J (2021). Development and characterization of infectious clones of two strains of Usutu virus. Virology.

[42] Ludwig GV, Iacono-Connors LC (1993). Insect-transmitted vertebrate viruses: flaviviridae. In Vitro Cell Dev Biol Anim.

[43] Boylan BT, Moreira FR, Carlson TW (2017). Mosquito cell-derived West Nile virus replicon particles mimic arbovirus inoculum and have reduced spread in mice. PLoS Negl Trop Dis.

[44] Fros JJ, Miesen P, Vogels CB, Gaibani P, Sambri V, Martina BE (2015). Comparative Usutu and West Nile virus transmission potential by local Culex pipiens mosquitoes in north-western Europe. One Health.

[45] Rückert C, Prasad AN, Garcia-Luna SM, Robison A, Grubaugh ND, Weger-Lucarelli J, Ebel GD (2019). Small RNA responses of Culex mosquitoes and cell lines during acute and persistent virus infection. Insect Biochem Mol Biol.

[46] Clé M, Constant O, Barthelemy J, Desmetz C, Martin MF, Lapeyre L (2021). Differential neurovirulence of Usutu virus lineages in mice and neuronal cells. J Neuroinflammation.

[47] Riccetti S, Sinigaglia A, Desole G, Nowotny N, Trevisan M, Barzon L (2020). Modelling West Nile Virus and Usutu Virus pathogenicity in human neural stem cells. Viruses.

[48] Pepin KM, Lambeth K, Hanley KA (2008). Asymmetric competitive suppression between strains of dengue virus. BMC Microbiol.

[49] Kenney JL, Solberg OD, Langevin SA, Brault AC (2014). Characterization of a novel insect-specific flavivirus from Brazil: potential for inhibition of infection of arthropod cells with medically important flaviviruses. J Gen Virol.

[50] Laureti M, Paradkar PN, Fazakerley JK, Rodriguez-Andres J (2020). Superinfection Exclusion in Mosquitoes and its potential as an Arbovirus Control Strategy. Viruses.

[51] Prow NA, Edmonds JH, Williams DT, Setoh YX, Bielefeldt-Ohmann H, Suen WW (2016). Virulence and evolution of West Nile Virus, Australia, 1960–2012. Emerg Infect Dis.

[52] Körsten C, AL-Hosary AA, Holicki CM, Schäfer M, Tews BA, Vasić A (2023). Simultaneous coinfections with West Nile Virus and Usutu Virus in Culex pipiens and Aedes vexans mosquitoes. Transbound Emerg Dis.

