# Supplementary material for: Cellular co-infections of West Nile virus and Usutu virus influence virus growth kinetics
Source: Virol J. 2023 Oct 13;20:234. doi: 10.1186/s12985-023-02206-9 (PMC10576383; doi:10.1186/s12985-023-02206-9)
Supplement: Supplementary file 1 — Supplementary material: additional file 1: Fig.S1. Cytopathic effects of WNV lineage 2 (Germany, 2018) in mammalian, avian and mosquito cell lines. Fig. S2. Growth kinetics of West Nile virus (WNV) and Usutu virus (USUV) on various cell lines. Fig. S3. Virus secretion in mono- and co-infections of WNV lineage 1 and USUV Europe 3. Fig. S4. Virus secretion in mono- and co-infections of WNV lineage 2 and USUV Africa 3. Fig. S5. Virus secretion in mono- and co-infections of WNV lineage 1 and USUV Africa 3. Table S1. Description of the cell lines and media used in the study. Table S2. Description of the virus stocks used in the study. Table S3. Table comparing standard curves of the synthetic and naïve standards for Usutu virus (USUV). Table S4. Table comparing standard curves of the synthetic and naïve standards for West Nile virus (WNV). Table S5. Dependency of viral replication on the virus strain, time point and the infected cell line. Table S6. Dependence of WNV replication on constellation of the respective WNV mono-infections and the time points. Table S7. Dependency of USUV viral replication on the cells at the different time points. Table S8. Comparison of virus titres examined by virus titration or RT-qPCR on Vero B4 cells. Table S9. Comparison of virus titres examined by virus titration or RT-qPCR on C6/36 cells. Table S10. Comparison of virus combinations at different time points for Vero B4 cells. Table S11. Dependency of viral replication on the multiplicity of infection and time point. Table S12. Dependency of USUV viral replication on the virus constellation and time points in CT cells. Table S13. Dependency of WNV viral replication on the virus constellation and time points in CT cells. Table S14. Dependence of virus combinations and different time points for C6/36 cells. Table S15. Dependency of viral replication on the virus constellation and the time points in GN-R. Table S16. Dependency of viral replication on virus constellation and time point in Ve [file 12985_2023_2206_MOESM1_ESM.docx]

Cellular co-infections of West Nile virus and Usutu virus influence virus growth kinetics

**Christin Körsten ^1, †^, Hannah Reemtsma ^2, †^, Ute Ziegler ^2^, Susanne Fischer ^1^, Birke A. Tews ^1^, Martin H. Groschup ^2^, Cornelia Silaghi ^1^, Ana Vasic ^1, a^, and Cora M. Holicki ^2,^ ***

^1^ Friedrich-Loeffler-Institut, Federal Research Institute for Animal Health, Institute of Infectology, 17493 Greifswald-Insel Riems, Germany; christin.koersten@fli.de (C.K.), susanne.fischer@fli.de (S.F.), birke.tews@fli.de (B.A.T.), cornelia.silaghi@fli.de (C.S.)

^2^ Friedrich-Loeffler-Institut, Federal Research Institute for Animal Health, Institute of Novel and Emerg-ing Infectious Diseases, 17493 Greifswald-Insel Riems, Germany; hannah.reemtsma@fli.de (H.R.); ute.ziegler@fli.de (U.Z.); martin.groschup@fli.de (M.H.G.)

^a^ Present address: Scientific Institute of Veterinary Medicine of Serbia, Belgrade, Serbia; ana.vasic@nivs.rs (A.V.)

^†^ Contributed equally and share first authorship

^*^ Correspondence: cora.holicki@fli.de

**Table S1. Description of the cell lines and media used in the study.**

| **Cell**  **line** | **Origin** | **Cultivation temperature** | **Cultivation medium** | **Concentration for growth curves (cells/mL)** | **Concentration for titration (cells/mL)** |
| --- | --- | --- | --- | --- | --- |
| Vero B4 | African green monkey  (*Chlorocebus sabaeus*)  Kidney; fibroblast | 37 °C | MEM supplemented with non-essential amino acids | 2 x 10^5^ | 1 x 10^5^ |
| GN-R | Domestic goose  (*Anser anser f. domestica*)  Kidney; fibroblast | 37 °C | Ham’s F12 and Iscove′s Modified Dulbecco′s Medium (IMDM) 1:1 | 2 x 10^5^ | n.d. |
| C6/36 | Asian tiger mosquito  *(Aedes albopictus)*  Larvae  [1] | 28 °C | MEM supplemented with non-essential amino acids and 25 mM HEPES | 5 x 10^5^ | n.d. |
| CT | Western encephalitis mosquito  *(Culex tarsalis)*  Embryo  [2] | 28 °C | Schneider’s Insect Medium supplemented with L-Gln, L-Asn, CaCl_2_, GSH, Insulin | 5 x 10^5^ | n.d. |

MEM: minimum essential medium; Gln: glutamine; Asn: Asparagine; GSH: glutathione; n.d. not done

References:

1. Singh KRP (1967) Cell cultures derived from larvae of *Aedes albopictus* (Skuse) and *Aedes aegypti* (L.). Curr Sci:506–508

2. Chao J, Ball GH (1976) Comparison of amino acid utilization by cell lines of *Culex tarsalis* and of *Culex pipiens*. Invertebrate Tissue Culture:263–266. https://doi.org/10.1016/B978-0-12-429740-1.50028-X

**Table S2. Description of the virus stocks used in the study.**

| **Name of virus** | **Lineage** | **Origin** | **GenBank accession no.** | **Publication** | **Passages** | **Titre in TCID_50_/mL (Vero B4)** |
| --- | --- | --- | --- | --- | --- | --- |
| USUV Europe 3 | USUV Europe 3 | Eurasian Blackbird  (*Turdus merula)*  Baden-Wuerttemberg, Germany  2011 | HE599647 | (Becker et al., 2012)[1] | 3 | 10^8.13^ |
| USUV Africa 3 | USUV Africa 3 | Eurasian Blackbird  (*Turdus merula)*  Saxony, Germany  2016 | KY084496 | [2] | 3 | 10^8.25^ |
| WNV  Italy | WNV lineage 1 | Human  (*Homo sapiens)*  Region Ferrara, Italy  2009 | HM991273/  HM641225 | Italy TOS 09  [3] | 7 | 10^8.88^ |
| WNV Austria | WNV lineage 2 | Goshawk  (*Accipiter gentilis*)  Weiz, Austria  2009 | HM015884 | [4] | 4 | 10^8.00^ |
| WNV Germany 2018 | WNV lineage 2 | Great grey owl  (*Strix nebulosa*),  Saxony Anhalt,  Germany  2018 | MH924836 | [5] | 3 | 10^9.29^ |
| WNV Germany 2019 | WNV lineage 2 | Eurasian golden plower  (*Pluvialis apricaria)*  Saxony Anhalt,  Germany  2019 | LR743431 | [6] | 2 | 10^9.38^ |

TCID_50_: tissue culture infective dose 50

References:

1. Becker N, Jöst H, Ziegler U. et al. (2012) Epizootic emergence of Usutu virus in wild and captive birds in Germany. PLoS One 2012, 7, e32604, doi:10.1371/journal.pone.0032604.

2. Sieg M, Schmidt V, Ziegler U et al. (2017) Outbreak and Cocirculation of Three Different Usutu Virus Strains in Eastern Germany. Vector Borne Zoonotic Dis. 2017, 17, 662–664, doi:10.1089/vbz.2016.2096.

3. Rossini G, Carletti F, Bordi L et al. (2011) Phylogenetic analysis of West Nile virus isolates, Italy, 2008-2009. Emerg. Infect. Dis. 2011, 17, 903–906, doi:10.3201/eid1705.101569.

4. Wodak E, Richter S, Bagó Z et al. (2012) Detection and molecular analysis of West Nile virus infections in birds of prey in the eastern part of Austria in 2008 and 2009. Vet Microbiol 149:358–366. https://doi.org/10.1016/j.vetmic.2010.12.012

5. Ziegler U, Lühken R, Keller M et al. (2019) West Nile virus epizootic in Germany, 2018. Antiviral Res 162:39–43. https://doi.org/10.1016/j.antiviral.2018.12.005

6. Ziegler U, Santos PD, Groschup MH et al. (2020) West Nile Virus Epidemic in Germany Triggered by Epizootic Emergence, 2019. Viruses 12:448. https://doi.org/10.3390/v12040448

**Table S3. Table comparing standard curves of the synthetic and naïve standards for Usutu virus (USUV)**

|  | **Synthetic USUV standard** | **Naïve standard USUV Europe 3** | | **Naïve standard USUV Africa 3** | |
| --- | --- | --- | --- | --- | --- |
| **Ct value** | **Copies/mL** | **Copies/mL** | **TCID_50_/mL** | **Copies/mL** | **TCID_50_/mL** |
| 20.4 | 5.18E+08 | 5.42E+08 | 1.35E+06 | 3.02E+08 | 1.78E+06 |
| 23.8 | 5.18E+07 | 3.94E+07 | 1.35E+05 | 2.90E+07 | 1.78E+05 |
| 27.0 | 5.18E+06 | 4.10E+06 | 1.35E+04 | 2.24E+06 | 1.78E+04 |
| 30.5 | 5.18E+05 | 2.90E+05 | 1.35E+03 | 2.34E+05 | 1.78E+03 |
| 33.4 | 5.18E+04 | 2.42E+04 | 1.35E+02 | 4.82E+04 | 1.78E+02 |

To estimate the virus concentrations (i.e., TCID_50_) from the RT-qPCR results the synthetic and corresponding naïve standard curves were used. The naïve standard curves were generated through the dilution of original virus stocks with a known titre (as determined by titration). Ct= cycle threshold.

**Table S4. Table comparing standard curves of the synthetic and naïve standards for West Nile virus (WNV)**

|  | **Synthetic WNV standard** | **Naïve standard WNV Germany 2018** | | **Naïve standard WNV Germany 2019** | |
| --- | --- | --- | --- | --- | --- |
| **Ct value** | **Copies/mL** | **Copies/mL** | **TCID_50_/mL** | **Copies/mL** | **TCID_50_/mL** |
| 20.0 | 1.00E+07 | 6.20E+06 | 2.00E+07 | 5.08E+06 | 2.30E+07 |
| 23.4 | 1.00E+06 | 4.44E+05 | 2.00E+06 | 5.62E+05 | 2.30E+06 |
| 26.6 | 1.00E+05 | 4.56E+04 | 2.00E+05 | 3.94E+04 | 2.30E+05 |
| 29.7 | 1.00E+04 | 4.78E+03 | 2.00E+04 | 3.22E+03 | 2.30E+04 |
| 32.0 | 1.00E+03 | 1.58E+03 | 2.00E+03 | 1.08E+03 | 2.30E+03 |

To estimate the virus concentrations (i.e., TCID_50_) from the RT-qPCR results the synthetic and corresponding naïve standard curves were used. The naïve standard curves were generated through the dilution of original virus stocks with a known titre (as determined by titration). Ct= cycle threshold.


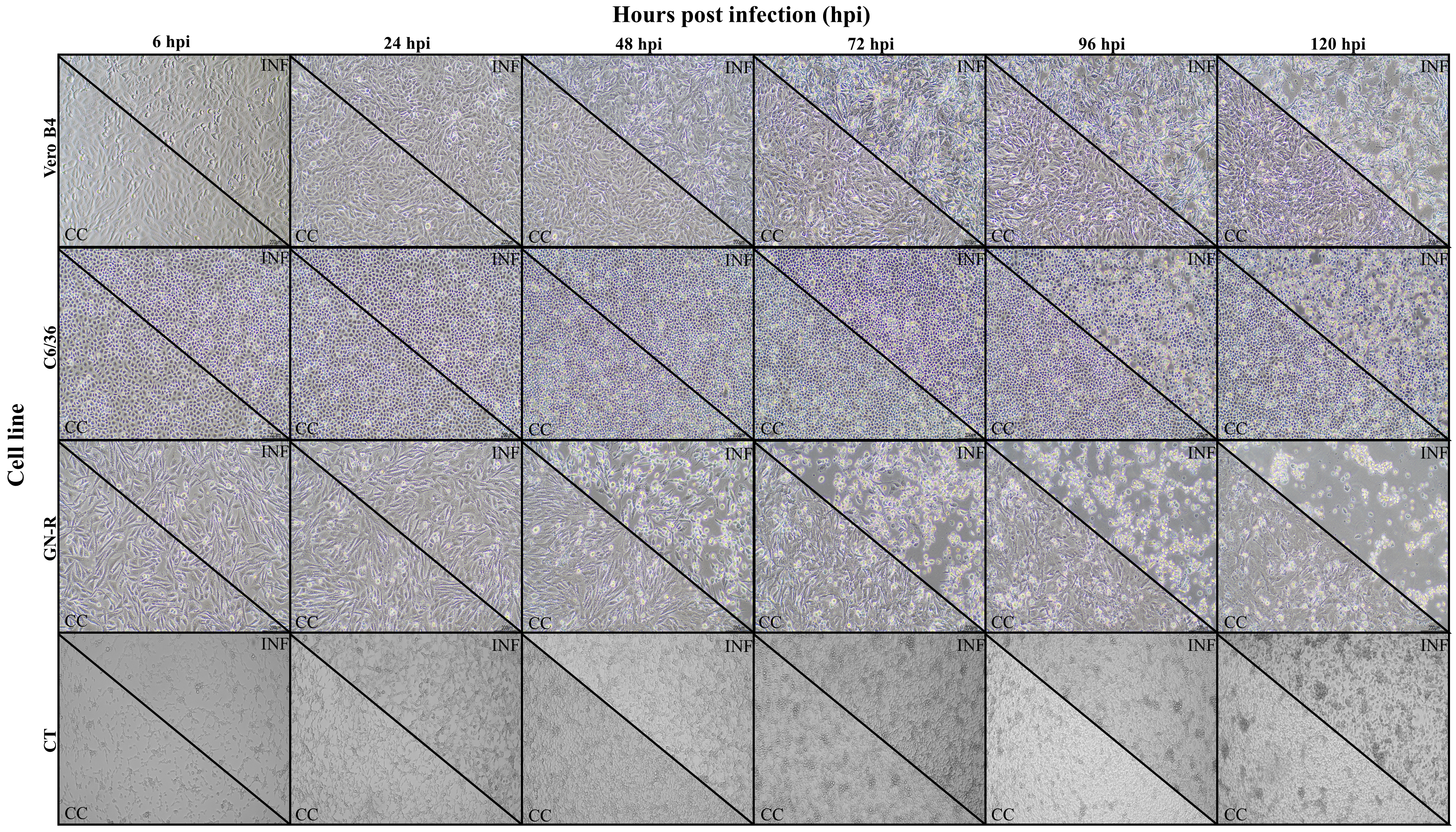


**Fig. S1** **Cytopathic effects of WNV lineage 2 (Germany, 2018) in mammalian, avian and mosquito cell lines.** In each quadrant the cell control (CC) is displayed on the bottom left and the top right the infected cell layer (INF). Photos were taken in the original magnification x100 (Microscope: Nikon Eclipse Ts2, Nikon Europe B.V., Amstelveen, Netherlands; Camera: DS-Fi3, Nikon Europe B.V., Amstelveen, Netherlands with phase contrast (for Vero B4, C6/36 and GN-R) and Axiocam 202 mono, Carl Zeiss, Oberkochen, Germany without phase contrast (for CT)).


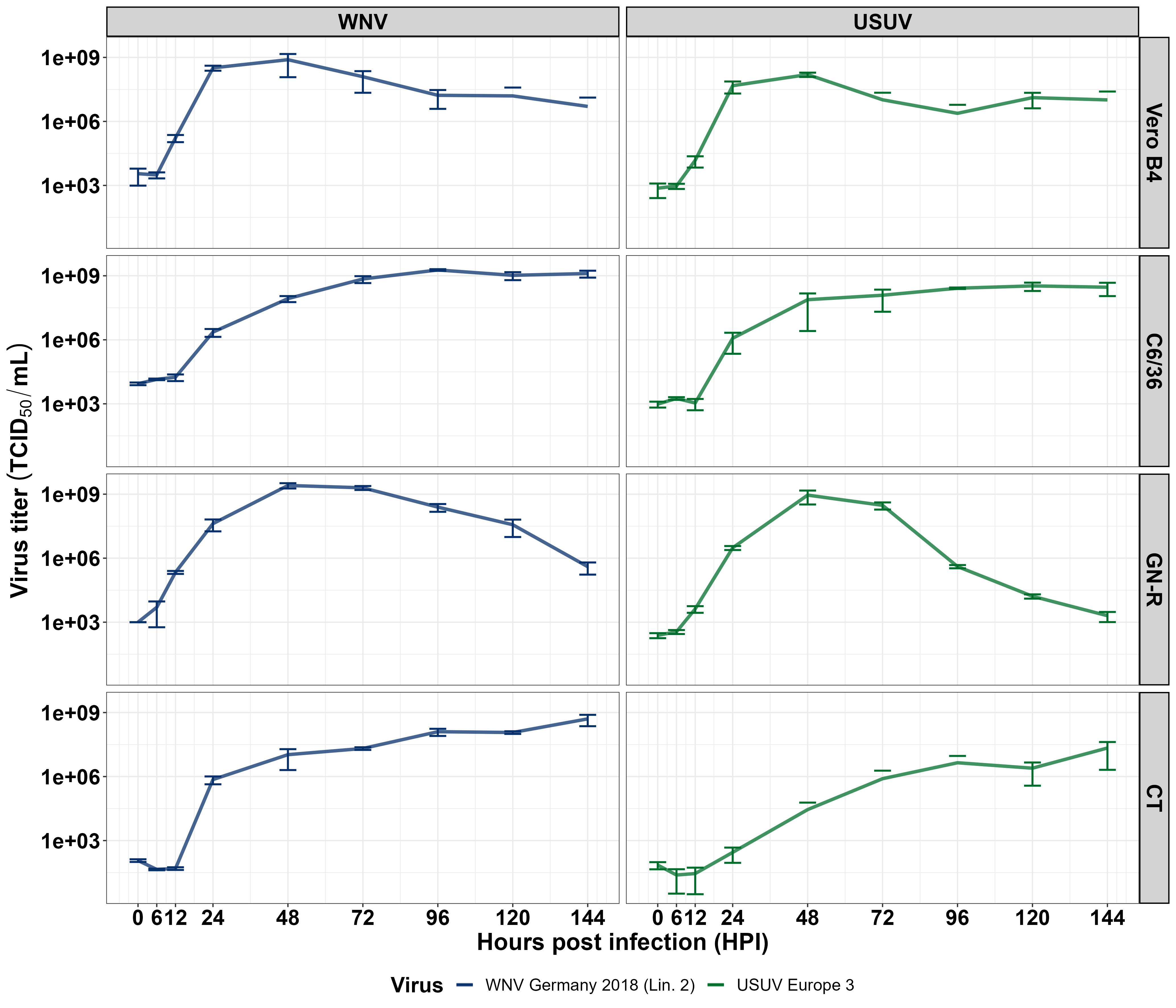


**Fig. S2: Growth kinetics of West Nile virus (WNV) and Usutu virus (USUV) on various cell lines.** Mono-infections were performed with WNV lineage 2 isolated in Germany in 2018 as well as USUV lineage Europe 3 isolated in Germany in 2011 in vertebrate (Vero B4 and GN-R) and mosquito cell lines (C6/36 and CT). All mono-infections were performed with a multiplicity of infection (MOI) of 1. The solid lines are drawn through the mean values of the three biological replicates for all tested time points. The error bars represent the standard deviation (± SD). Incomplete error bars occur when y-min of the error bars is negative and therefore not displayed in the logarithmic y-scale.

**
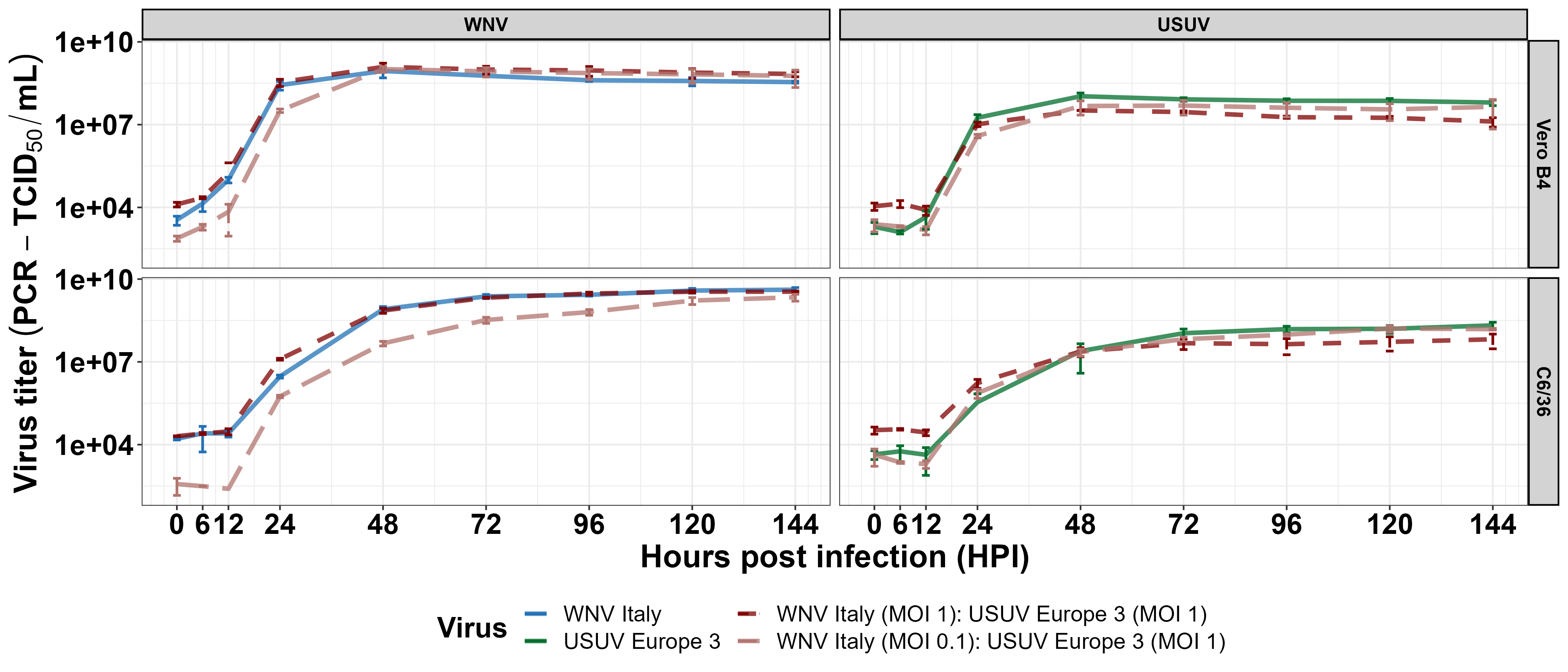
** **Fig. S3: Virus secretion in mono- and co-infections of WNV lineage 1 and USUV Europe 3.** Mono- and co-infections were performed with WNV lineage 1 isolated in Italy in 2009 and USUV Europe 3 isolated in Germany in 2011 in a vertebrate (Vero B4) as well as a mosquito cell line (C6/36). Co-infections were performed with a multiplicity of infection (MOI) of 1 for USUV and either 1 or 0.1 for WNV. The solid and dashed lines are drawn through the mean values of the three biological replicates for all tested time points measured by RT-qPCR based on a relative and absolute standard curve running in parallel. The error bars represent the standard deviation around the mean (± SD). Incomplete error bars occur when y-min of the error bars is negative and therefore not displayed in the logarithmic y-scale.

**
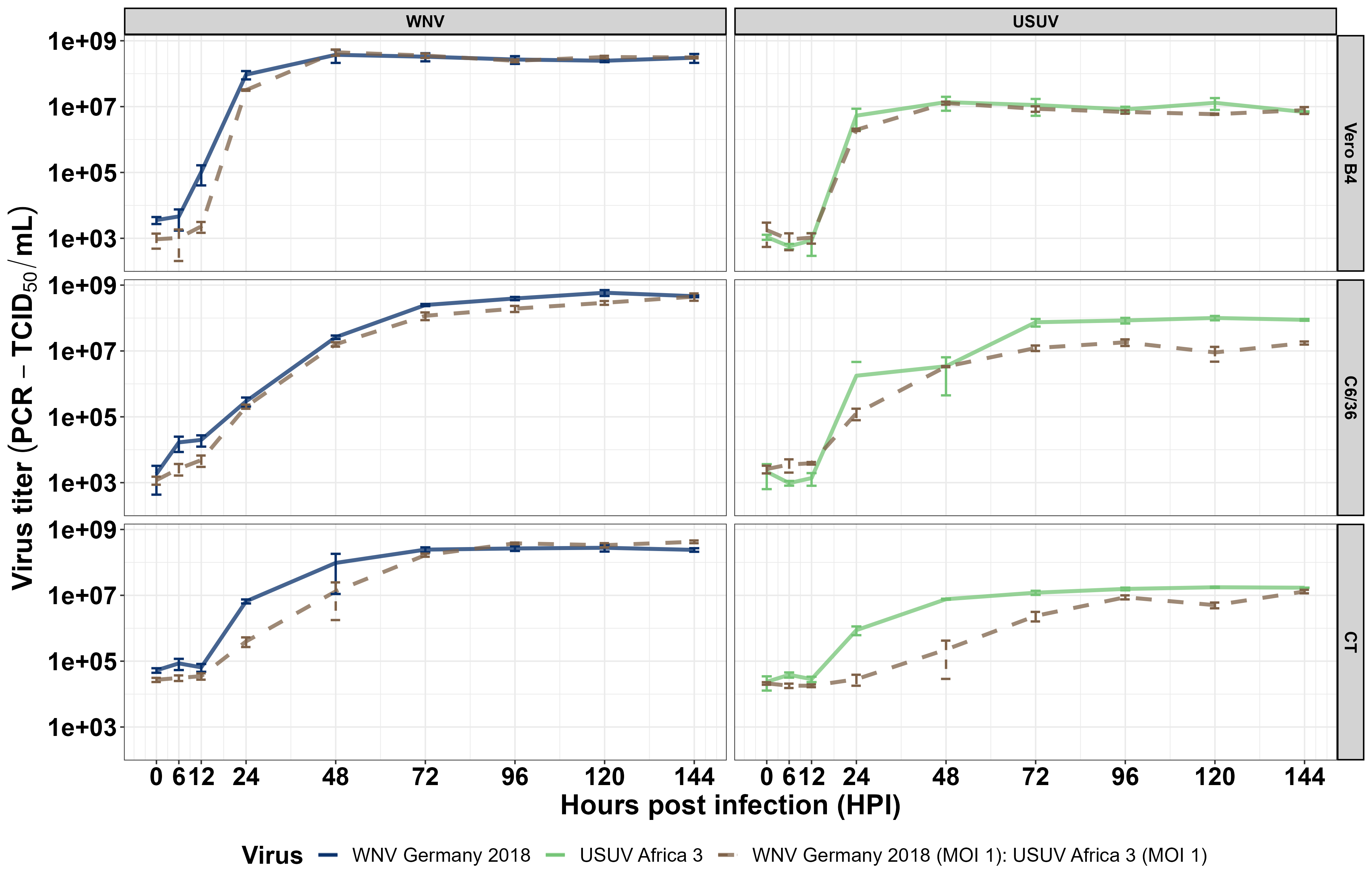
 Fig. S4: Virus secretion in mono- and co-infections of WNV lineage 2 and USUV Africa 3.** Mono- and co-infections were performed with WNV lineage 2 isolated in Germany in 2018 and USUV Africa 3 isolated in Germany in 2016 in a vertebrate (Vero B4) as well as mosquito cell lines (C6/36 and CT). Co-infections were performed with a multiplicity of infection (MOI) of 1 for both USUV and WNV. The solid and dashed lines are drawn through the mean values of the three biological replicates for all tested time points measured by RT-qPCR based on a relative and absolute standard curve running in parallel. The error bars represent the standard deviation around the mean (± SD). Incomplete error bars occur when y-min of the error bars is negative and therefore not displayed in the logarithmic y-scale.

**
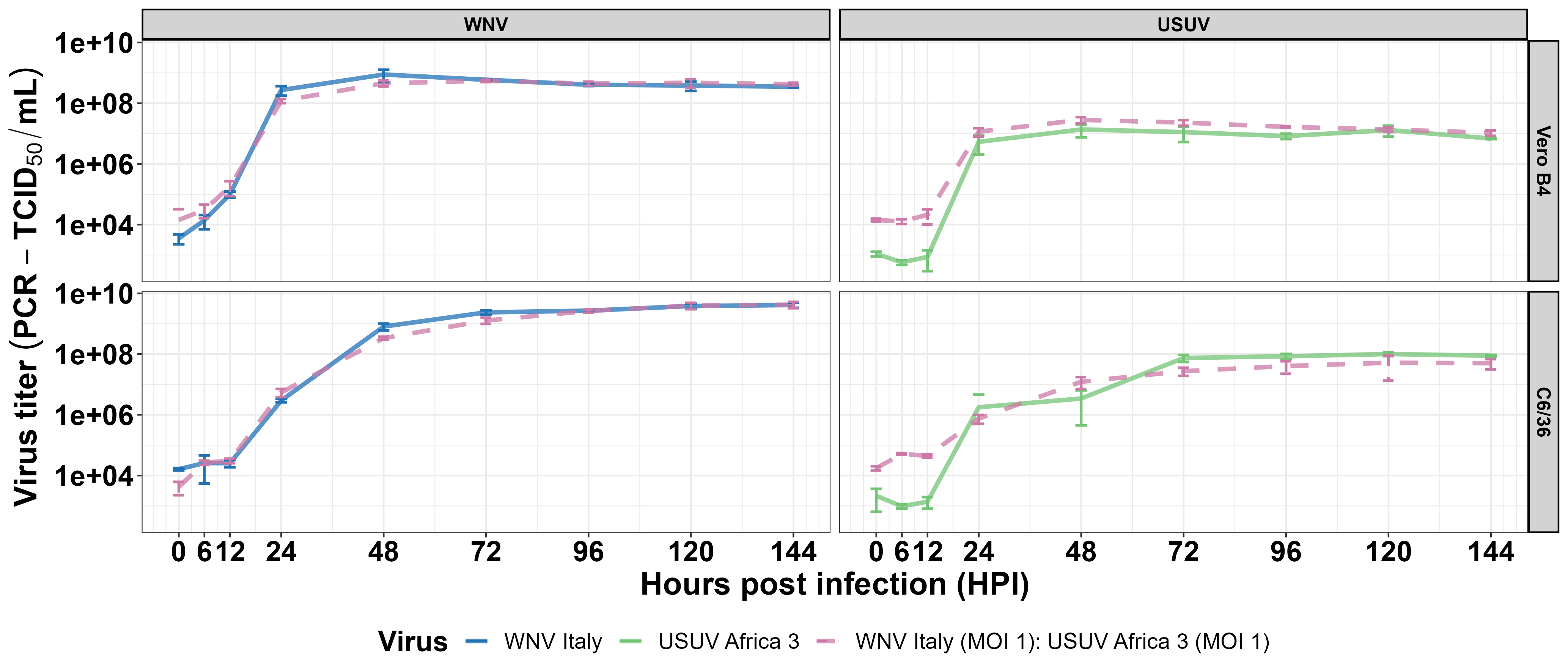
**

**Fig. S5: Virus secretion in mono- and co-infections of WNV lineage 1 and USUV Africa 3.** Mono- and co-infections were performed with WNV lineage 1 isolated in Italy in 2009 and USUV Africa 3 isolated in Germany in 2016 in a vertebrate (Vero B4) as well as a mosquito cell line (C6/36). Co-infections were performed with a multiplicity of infection (MOI) of 1 for both USUV and WNV. The solid and dashed lines are drawn through the mean values of the three biological replicates for all tested time points measured by RT-qPCR based on a relative and absolute standard curve running in parallel. The error bars represent the standard deviation around the mean (± SD). Incomplete error bars occur when y-min of the error bars is negative and therefore not displayed in the logarithmic y-scale.

**Table S5. Dependency of viral replication on the virus strain, time point and the infected cell line.**

| **Viral genome copies** | **Factors** | **Df** | **Sum.Sq** | **Mean.Sq** | **F.value** | **Pr.(>F.)** |
| --- | --- | --- | --- | --- | --- | --- |
| WNV | Virus | 3 | 6.41 | 2.135 | 62.461 | **< 2e-16 ***** |
| WNV | Time point | 2 | 31.92 | 15.960 | 466.845 | **< 2e-16 ***** |
| WNV | Cell line | 1 | 9.35 | 9.351 | 273.527 | **< 2e-16 ***** |
| WNV | Virus:Cell line | 3 | 1.68 | 0.559 | 16.344 | **2.06e-07 ***** |
| WNV | Virus:Time point | 6 | 1.63 | 0.272 | 7.958 | **6.02e-06 ***** |
| WNV | Time point:Cell line | 2 | 15.13 | 7.564 | 221.248 | **< 2e-16 ***** |
| WNV | Virus:Time point:Cell line | 6 | 0.60 | 0.100 | 2.933 | 0.0163 * |
| WNV | Residuals | 47 | 1.61 | 0.034 |  |  |
| USUV | Virus | 1 | 2.757 | 2.757 | 10.862 | **0.003043 **** |
| USUV | Time point | 2 | 14.008 | 7.004 | 27.590 | **6.01e-07 ***** |
| USUV | Cell line | 1 | 3.669 | 3.669 | 14.453 | **0.000868 ***** |
| USUV | Virus:Cell line | 1 | 0.569 | 0.569 | 2.242 | 0.147304 |
| USUV | Virus:Time point | 2 | 1.123 | 0.561 | 2.211 | 0.131393 |
| USUV | Time point:Cell line | 2 | 6.150 | 3.075 | 12.113 | **0.000231 ***** |
| USUV | Virus:Time point:Cell line | 2 | 0.548 | 0.274 | 1.080 | 0.355548 |
| USUV | Residuals | 24 | 6.093 | 0.254 |  |  |

ANOVA test showing dependency of viral replication on the virus strain, time point post infection and the infected cell line (Vero B4 and C6/36) (based on Figure 2). Term significant at alpha = 0.05; *p<0.1, **p<0.01, ***p<0.001. To explore the relation between viral genome copies of co-infections, an ANOVA model (Viral genome copies=virus+time+cell.line+virus*time*cell.line) was performed using R (v3.6.2, x64). WNV and USUV viral replication is dependent on the virus strain, the time point and the infected cell line. For WNV the interaction between all three factors (Strain, time point, cell line) is significant and therefore all scenarios were tested individually. A highly statistically significant (p<0.0001) difference in the viral replication stretching all three time points was observed on the C6/36 between the virus strains WNV Germany 2018 and WNV Germany 2019 or WNV Italy 2009, respectively. For USUV, only the interaction between the cell line and the time point was significant.

**Table S6. Dependence of WNV replication on constellation of the respective WNV mono-infections and the time points.**

|  | **Viral genome copies** | **Effect** | **Estimate** | **SE** | **Df** | **t.ratio** | **p.value** |
| --- | --- | --- | --- | --- | --- | --- | --- |
| 24 | Vero B4 | WNV Austria effect - WNV Germany 2018 effect | 0.3416 | 0.151 | 47 | 2.263 | 0.1216 |
| 24 | Vero B4 | WNV Austria effect - WNV Germany 2019 effect | 0.1243 | 0.151 | 47 | 0.824 | 0.8430 |
| 24 | Vero B4 | WNV Austria effect - WNV I Italy TOS-09 effect | -0.1150 | 0.151 | 47 | -0.762 | 0.8712 |
| 24 | Vero B4 | WNV Germany 2018 effect - WNV Germany 2019 effect | -0.2173 | 0.151 | 47 | -1.439 | 0.4817 |
| 24 | Vero B4 | WNV Germany 2018 effect - WNV I Italy TOS-09 effect - | -0.4566 | 0.151 | 47 | -3.025 | 0.0203 * |
| 24 | Vero B4 | WNV Germany 2019 effect - WNV I Italy TOS-09 effect | -0.2393 | 0.151 | 47 | -1.585 | 0.3967 |
| 48 | Vero B4 | WNV Austria effect - WNV Germany 2018 effect | -0.0498 | 0.151 | 47 | -0.330 | 0.9875 |
| 48 | Vero B4 | WNV Austria effect - WNV Germany 2019 effect | -0.6143 | 0.151 | 47 | -4.069 | 0.0010 ******* |
| 48 | Vero B4 | WNV Austria effect - WNV I Italy TOS-09 effect | -0.4207 | 0.151 | 47 | -2.786 | 0.0371 ***** |
| 48 | Vero B4 | WNV Germany 2018 effect - WNV Germany 2019 effect | -0.5646 | 0.151 | 47 | -3.740 | 0.0027 ****** |
| 48 | Vero B4 | WNV Germany 2018 effect - WNV I Italy TOS-09 effect - | -0.3709 | 0.151 | 47 | -2.457 | 0.0803 ***** |
| 48 | Vero B4 | WNV Germany 2019 effect - WNV I Italy TOS-09 effect | 0.1937 | 0.151 | 47 | 1.283 | 0.5782 |
| 72 | Vero B4 | WNV Austria effect - WNV Germany 2018 effect | -0.1127 | 0.151 | 47 | -0.747 | 0.8776 |
| 72 | Vero B4 | WNV Austria effect - WNV Germany 2019 effect | -0.4160 | 0.151 | 47 | -2.756 | 0.0401 ***** |
| 72 | Vero B4 | WNV Austria effect - WNV I Italy TOS-09 effect | -0.3783 | 0.151 | 47 | -2.506 | 0.0720 ***** |
| 72 | Vero B4 | WNV Germany 2018 effect - WNV Germany 2019 effect | -0.3033 | 0.151 | 47 | -2.009 | 0.1994 |
| 72 | Vero B4 | WNV Germany 2018 effect - WNV I Italy TOS-09 effect - | -0.2656 | 0.151 | 47 | -1.759 | 0.3056 |
| 72 | Vero B4 | WNV Germany 2019 effect - WNV I Italy TOS-09 effect | 0.0377 | 0.151 | 47 | 0.250 | 0.9945 |
| 24 | C6/36 | WNV Austria effect - WNV Germany 2018 effect | 1.1934 | 0.151 | 47 | 7.905 | <.0001 ******* |
| 24 | C6/36 | WNV Austria effect - WNV Germany 2019 effect | 0.1633 | 0.151 | 47 | 1.082 | 0.7022 |
| 24 | C6/36 | WNV Austria effect - WNV I Italy TOS-09 effect | 0.1810 | 0.151 | 47 | 1.199 | 0.6306 |
| 24 | C6/36 | WNV Germany 2018 effect - WNV Germany 2019 effect | -1.0301 | 0.151 | 47 | -6.823 | <.0001 ******* |
| 24 | C6/36 | WNV Germany 2018 effect - WNV I Italy TOS-09 effect - | -1.0124 | 0.151 | 47 | -6.706 | <.0001 ******* |
| 24 | C6/36 | WNV Germany 2019 effect - WNV I Italy TOS-09 effect | 0.0177 | 0.151 | 47 | 0.117 | 0.9994 |
| 48 | C6/36 | WNV Austria effect - WNV Germany 2018 effect | 0.9122 | 0.151 | 47 | 6.042 | <.0001 ******* |
| 48 | C6/36 | WNV Austria effect - WNV Germany 2019 effect | -0.3370 | 0.151 | 47 | -2.232 | 0.1294 |
| 48 | C6/36 | WNV Austria effect - WNV I Italy TOS-09 effect | -0.5718 | 0.169 | 47 | -3.388 | 0.0076 ****** |
| 48 | C6/36 | WNV Germany 2018 effect - WNV Germany 2019 effect | -1.2492 | 0.151 | 47 | -8.275 | <.0001 ******* |
| 48 | C6/36 | WNV Germany 2018 effect - WNV I Italy TOS-09 effect - | -1.4840 | 0.169 | 47 | -8.792 | <.0001 ******* |
| 48 | C6/36 | WNV Germany 2019 effect - WNV I Italy TOS-09 effect | -0.2348 | 0.169 | 47 | -1.391 | 0.5110 |
| 72 | C6/36 | WNV Austria effect - WNV Germany 2018 effect | -0.0176 | 0.151 | 47 | -0.117 | 0.9994 |
| 72 | C6/36 | WNV Austria effect - WNV Germany 2019 effect | -0.9540 | 0.151 | 47 | -6.319 | <.0001 ******* |
| 72 | C6/36 | WNV Austria effect - WNV I Italy TOS-09 effect | -0.9923 | 0.151 | 47 | -6.573 | <.0001 ******* |
| 72 | C6/36 | WNV Germany 2018 effect - WNV Germany 2019 effect | -0.9364 | 0.151 | 47 | -6.203 | <.0001 ******* |
| 72 | C6/36 | WNV Germany 2018 effect - WNV I Italy TOS-09 effect - | -0.9747 | 0.151 | 47 | -6.457 | <.0001 ******* |
| 72 | C6/36 | WNV Germany 2019 effect - WNV I Italy TOS-09 effect | -0.0383 | 0.151 | 47 | -0.254 | 0.9942 |

ANOVA with PostHoc test (Least-Squares Means) showing the dependence of WNV virus replication on the viral constellation of the respective WNV mono-infections and the time points post infection in infected Vero B4 and C6/36 cells (based on Figure 2). P-value adjustment was conducted using the tukey method. Term significant at alpha = 0.05; *p<0.1, **p<0.01, ***p<0.001.

**Table S7. Dependency of USUV viral replication on the cells at the different time points.**

| **Time point** | **USUV lineage** | **Contrast** | **Estimate** | **SE** | **Df** | **t.ratio** | **p.value** |
| --- | --- | --- | --- | --- | --- | --- | --- |
| 24 | Europe 3 | C636 effect - VeroB4 effect | -1.973 | 0.411 | 24 | -4.795 | 0.0001 ******* |
| 24 | Africa 3 | C636 effect - VeroB4 effect | -1.029 | 0.411 | 24 | -2.501 | 0.0196 ***** |
| 48 | Europe 3 | C636 effect - VeroB4 effect | -0.798 | 0.411 | 24 | -1.939 | 0.0643 ***** |
| 48 | Africa 3 | C636 effect - VeroB4 effect | -0.984 | 0.411 | 24 | -2.392 | 0.0250 ***** |
| 72 | Europe 3 | C636 effect - VeroB4 effect | 0.100 | 0.411 | 24 | 0.244 | 0.8091 |
| 72 | Africa 3 | C636 effect - VeroB4 effect | 0.852 | 0.411 | 24 | 2.071 | 0.0493 ***** |

ANOVA with PostHoc test (Least-Squares Means) showing dependency of USUV viral replication on the cells (Vero B4 and C6/36) at the different time points after infection with USUV Europe 3 and Africa 3, respectively (based on Figure 2). P-value adjustment was conducted using the tukey method. Term significant at alpha = 0.05; *p<0.1, **p<0.01, ***p<0.001.

**Table S8. Comparison of virus titres examined by virus titration or RT-qPCR on Vero B4 cells.**

| **Time point** | **Virus lineage** | **Contrast** | **Estimate** | | **SE** | **Df** | **t.ratio** | **p.value** |
| --- | --- | --- | --- | --- | --- | --- | --- | --- |
| 24 | WNV Germany 2018 | PCR effect - Titration effect | | -0.5396 | 0.408 | Inf | -1.323 | 0.1857 |
| 48 | WNV Germany 2018 | PCR effect - Titration effect | | -0.2436 | 0.429 | Inf | -0.567 | 0.5706 |
| 72 | WNV Germany 2018 | PCR effect - Titration effect | | 0.5252 | 0.408 | Inf | 1.286 | 0.1984 |
| 24 | WNV Germany 2019 | PCR effect - Titration effect | | -0.00983 | 0.281 | Inf | -0.035 | 0.9721 |
| 48 | WNV Germany 2019 | PCR effect - Titration effect | | 1.07517 | 0.295 | Inf | 3.641 | 0.0003 ******* |
| 72 | WNV Germany 2019 | PCR effect - Titration effect | | 1.70350 | 0.275 | Inf | 6.195 | <.0001 ******* |
| 24 | WNV Austria | PCR effect - Titration effect | | -0.479 | 0.340 | Inf | -1.411 | 0.1584 |
| 48 | WNV Austria | PCR effect - Titration effect | | -0.543 | 0.349 | Inf | -1.557 | 0.1194 |
| 72 | WNV Austria | PCR effect - Titration effect | | 0.287 | 0.328 | Inf | 0.877 | 0.3807 |
| 24 | WNV Italy 2009 | PCR effect - Titration effect | | 0.1253 | 0.421 | Inf | 0.298 | 0.7659 |
| 48 | WNV Italy 2009 | PCR effect - Titration effect | | 0.7732 | 0.430 | Inf | 1.797 | 0.0724 ***** |
| 72 | WNV Italy 2009 | PCR effect - Titration effect | | 2.0929 | 0.393 | Inf | 5.330 | <.0001 ******* |
| 24 | USUV Europe 3 | PCR effect - Titration effect | | -0.392 | 0.525 | Inf | -0.746 | 0.4556 |
| 48 | USUV Europe 3 | PCR effect - Titration effect | | -0.173 | 0.572 | Inf | -0.303 | 0.7619 |
| 72 | USUV Europe 3 | PCR effect - Titration effect | | -0.118 | 0.520 | Inf | 2.229 | 0.0258 ***** |
| 24 | USUV Africa 3 | PCR effect - Titration effect | | -0.888 | 0.239 | Inf | -3.708 | 0.0002 ******* |
| 48 | USUV Africa 3 | PCR effect - Titration effect | | -0.370 | 0.246 | Inf | -1.502 | 0.1331 |
| 72 | USUV Africa 3 | PCR effect - Titration effect | | 0.219 | 0.233 | Inf | 0.938 | 0.3484 |

ANOVA with PostHoc test (Least-Squares Means) showing the comparison of virus titres examined by virus titration or RT-qPCR on Vero B4 cells at the time points 24, 48, and 72 h after infection with all six different virus strains. P-value adjustment was conducted using the tukey method. Term significant at alpha = 0.05; *p<0.1, **p<0.01, ***p<0.001.

**Table S9. Comparison of virus titres examined by virus titration or RT-qPCR on C6/36 cells.**

| **Time point** | **Virus lineage** | **Contrast** | **Estimate** | | **SE** | **Df** | **t.ratio** | **p.value** |
| --- | --- | --- | --- | --- | --- | --- | --- | --- |
| 24 | WNV Germany 2018 | PCR effect - Titration effect | | -0.8774 | 0.1607 | Inf | -5.458 | <.0001 ******* |
| 48 | WNV Germany 2018 | PCR effect - Titration effect | | -0.5002 | 0.2086 | Inf | -2.398 | 0.0165 ***** |
| 72 | WNV Germany 2018 | PCR effect - Titration effect | | -0.4381 | 0.2343 | Inf | -1.869 | 0.0616 ***** |
| 24 | WNV Germany 2019 | PCR effect - Titration effect | | 0.17350 | 0.173 | Inf | 1.001 | 0.3170 |
| 48 | WNV Germany 2019 | PCR effect - Titration effect | | 0.00942 | 0.173 | Inf | 0.054 | 0.9567 |
| 72 | WNV Germany 2019 | PCR effect - Titration effect | | 0.41500 | 0.173 | Inf | 2.393 | 0.0167 ***** |
| 24 | WNV Austria | PCR effect - Titration effect | | -0.663 | 0.286 | Inf | - 2.061 | 0.0393 ***** |
| 48 | WNV Austria | PCR effect - Titration effect | | -0.734 | 0.356 | Inf | -1.557 | 0.1194 |
| 72 | WNV Austria | PCR effect - Titration effect | | -0.914 | 0.362 | Inf | -2.526 | 0.0115 ***** |
| 24 | WNV Italy 2009 | PCR effect - Titration effect | | -0.209 | 0.155 | Inf | -1.345 | 0.1787 |
| 48 | WNV Italy 2009 | PCR effect - Titration effect | | 0.182 | 0.190 | Inf | 0.956 | 0.3391 |
| 72 | WNV Italy 2009 | PCR effect - Titration effect | | 0.703 | 0.155 | Inf | 4.530 | <.0001 ******* |
| 24 | USUV Europe 3 | PCR effect - Titration effect | | -0.609 | 0.299 | Inf | -2.034 | 0.0420 ***** |
| 48 | USUV Europe 3 | PCR effect - Titration effect | | -0.429 | 0.399 | Inf | -1.077 | 0.2817 |
| 72 | USUV Europe 3 | PCR effect - Titration effect | | 0.202 | 0.425 | Inf | 0.476 | 0.6343 |
| 24 | USUV Africa 3 | PCR effect - Titration effect | | -0.2295 | 0.305 | Inf | -0.753 | 0.4514 |
| 48 | USUV Africa 3 | PCR effect - Titration effect | | -1.3333 | 0.364 | Inf | -3.666 | 0.0002 ******* |
| 72 | USUV Africa 3 | PCR effect - Titration effect | | -0.5336 | 0.433 | Inf | -1.231 | 0.2183 |

ANOVA with PostHoc test (Least-Squares Means) showing the comparison of virus titres examined by virus titration or RT-qPCR on C6/36 cells at the time points 24, 48, and 72 h after infection with all six different virus strains. P-value adjustment was conducted using the tukey method. Term significant at alpha = 0.05; *p<0.1, **p<0.01, ***p<0.001.

**Table S10. Comparison of virus combinations at different time points for Vero B4 cells.**

| **Viral genome copies** | **Time point** | **Contrast** | **estimate** | | **SE** | | **Df** | | **t.ratio** | | **p.value** |
| --- | --- | --- | --- | --- | --- | --- | --- | --- | --- | --- | --- |
| WNV | 24 | WNV Germany 18 effect – USUV Europe 3+WNV Germany 18 0.1 effect | 1.14061 | 0.0876 | | 18 | | 13.015 | | <.0001 ******* | |
| WNV | 24 | WNV Germany 18 effect – USUV Europe 3+WNV Germany 18 1 effect | 0.0932 | 0.0876 | | 18 | | 1.064 | | 0.548 | |
| WNV | 24 | USUV Europe 3+WNV Germany 18 0.1 effect+ USUV Europe 3+WNV Germany 18 1 effect | -1.0474 | 0.0876 | | 18 | | -11.952 | | <.0001 ******* | |
| WNV | 48 | WNV Germany 18 effect – USUV Europe 3+WNV Germany 18 0.1 effect | 0.32699 | 0.0876 | | 18 | | 3.731 | | 0.0041 ****** | |
| WNV | 48 | WNV Germany 18 effect – USUV Europe 3+WNV Germany 18 1 effect | 0.23228 | 0.0876 | | 18 | | 2.65 | | 0.0411 ****** | |
| WNV | 48 | USUV Europe 3+WNV Germany 18 0.1 effect+ USUV Europe 3+WNV Germany 18 1 effect | -0.09472 | 0.0876 | | 18 | | -1.081 | | 0.5377 | |
| WNV | 72 | WNV Germany 18 effect – USUV Europe 3+WNV Germany 18 0.1 effect | 0.44188 | 0.0876 | | 18 | | 5.042 | | 0.0002 ******* | |
| WNV | 72 | WNV Germany 18 effect – USUV Europe 3+WNV Germany 18 1 effect | 0.4341 | 0.0876 | | 18 | | 4.953 | | 0.0003 ******* | |
| WNV | 72 | USUV Europe 3+WNV Germany 18 0.1 effect+ USUV Europe 3+WNV Germany 18 1 effect | -0.00779 | 0.0876 | | 18 | | -0.089 | | 0.9957 | |
| USUV | 24 | USUV Europe 3 effect – USUV Europe 3+WNV Germany 18 1 effect | 0.562 | 0.0687 | | 18 | | 8.183 | | <.0001 ******* | |
| USUV | 24 | USUV Europe 3 effect – USUV Europe 3+WNV Germany 18 0.1 effect | 0.445 | 0.0687 | | 18 | | 6.47 | | <.0001 ******* | |
| USUV | 24 | USUV Europe 3+WNV Germany 18 0.1 effect+ USUV Europe 3+WNV Germany 18 1 effect | -0.118 | 0.0687 | | 18 | | -1.713 | | 0.2276 | |
| USUV | 48 | USUV Europe 3 effect – USUV Europe 3+WNV Germany 18 1 effect | 0.3 | 0.0687 | | 18 | | 4.366 | | 0.001 ****** | |
| USUV | 48 | USUV Europe 3 effect – USUV Europe 3+WNV Germany 18 0.1 effect | 0.879 | 0.0687 | | 18 | | 12.791 | | <.0001 ******* | |
| USUV | 48 | USUV Europe 3+WNV Germany 18 0.1 effect+ USUV Europe 3+WNV Germany 18 1 effect | 0.579 | 0.0687 | | 18 | | 8.424 | | <.0001 ******* | |
| USUV | 72 | USUV Europe 3 effect – USUV Europe 3+WNV Germany 18 1 effect | 0.376 | 0.0687 | | 18 | | 5.47 | | 0.0001 ******* | |
| USUV | 72 | USUV Europe 3 effect – USUV Europe 3+WNV Germany 18 0.1 effect | 0.739 | 0.0687 | | 18 | | 10.762 | | <.0001 ******* | |
| USUV | 72 | USUV Europe 3+WNV Germany 18 0.1 effect+ USUV Europe 3+WNV Germany 18 1 effect | 0.364 | 0.0687 | | 18 | | 5.292 | | 0.0001 ******* | |

ANOVA with PostHoc test (Least-Squares Means) showing the comparison of virus combinations at different time points for Vero B4 cells (based on Figure 3). P-value adjustment was conducted using the tukey method for comparing a family of 3 estimates. Term significant at alpha = 0.05; *p<0.1, **p<0.01, ***p<0.001.

**Table S11. Dependency of viral replication on the multiplicity of infection and time point.**

| **Cell line** | **Viral genome copies** | **Factors** | **Df** | **Sum.Sq** | **Mean.Sq** | **F.value** | **Pr.(>F.)** |
| --- | --- | --- | --- | --- | --- | --- | --- |
| Vero B4 | WNV | MOI | 2 | 1.848 | 0.9242 | 80.23 | 1.08e-09 *** |
| Vero B4 | WNV | Time point | 2 | 3.368 | 1.6839 | 146.17 | 7.43e-12 *** |
| Vero B4 | WNV | MOI:Time point | 4 | 1.112 | 0.2780 | 24.13 | 5.03e-07 *** |
| Vero B4 | WNV | Residuals | 18 | 0.207 | 0.0115 |  |  |
| Vero B4 | USUV | MOI | 2 | 2.1557 | 1.0778 | 152.24 | 5.26e-12 *** |
| Vero B4 | USUV | Time point | 2 | 2.7892 | 1.3946 | 196.98 | 5.80e-13 *** |
| Vero B4 | USUV | MOI:Time point | 4 | 0.3891 | 0.0973 | 13.74 | 2.64e-05 *** |
| Vero B4 | USUV | Residuals | 18 | 0.1274 | 0.0071 |  |  |
| C6/36 | WNV | MOI | 2 | 4.78 | 2.391 | 138.253 | 1.19e-11 *** |
| C6/36 | WNV | Time point | 2 | 42.96 | 21.478 | 1241.941 | < 2e-16 *** |
| C6/36 | WNV | MOI:Time point | 4 | 0.06 | 0.015 | 0.842 | 0.517 |
| C6/36 | WNV | Residuals | 18 | 0.31 | 0.017 |  |  |
| C6/36 | USUV | MOI | 2 | 0.338 | 0.169 | 1.729 | 0.206 |
| C6/36 | USUV | Time point | 2 | 24.801 | 12.400 | 126.723 | 2.48e-11 *** |
| C6/36 | USUV | MOI:Time point | 4 | 0.525 | 0.131 | 1.341 | 0.293 |
| C6/36 | USUV | Residuals | 18 | 1.761 | 0.098 |  |  |
| GN-R | WNV | MOI | 2 | 3.428 | 1.714 | 282.85 | 2.52e-14 *** |
| GN-R | WNV | Time point | 2 | 19.065 | 9.532 | 1572.92 | < 2e-16 *** |
| GN-R | WNV | MOI:Time point | 4 | 1.269 | 0.317 | 52.33 | 1.14e-09 *** |
| GN-R | WNV | Residuals | 18 | 0.109 | 0.006 |  |  |
| GN-R | USUV | MOI | 2 | 26.736 | 13.368 | 2271.85 | < 2e-16 *** |
| GN-R | USUV | Time point | 2 | 11.714 | 5.857 | 995.42 | < 2e-16 *** |
| GN-R | USUV | MOI:Time point | 4 | 1.708 | 0.427 | 72.57 | 7.47e-11 *** |
| GN-R | USUV | Residuals | 18 | 0.106 | 0.006 |  |  |
| CT | WNV | MOI | 2 | 2.139 | 1.069 | 34.482 | 6.97e-07 *** |
| CT | WNV | Time point | 2 | 19.844 | 9.922 | 319.920 | 8.60e-15 *** |
| CT | WNV | MOI:Time point | 4 | 0.245 | 0.061 | 1.978 | 0.141 |
| CT | WNV | Residuals | 18 | 0.558 | 0.031 |  |  |
| CT | USUV | MOI | 2 | 0.517 | 0.259 | 161.3 | 3.22e-12 *** |
| CT | USUV | Time point | 2 | 24.760 | 12.380 | 7716.9 | < 2e-16 *** |
| CT | USUV | MOI:Time point | 4 | 0.863 | 0.216 | 134.4 | 3.81e-13 *** |
| CT | USUV | Residuals | 18 | 0.029 | 0.002 |  |  |

ANOVA test showing dependency of viral replication on the multiplicity of infection and time point post infection for each infected cell line (based on Figure 3). Term significant at alpha = 0.05; *p<0.1, **p<0.01, ***p<0.001. To explore the relation between viral genome copies of co-infections, an ANOVA model (Viral genome copies= virus+time+virus*time) was performed using R (v3.6.2, x64). In the co-infection between WNV Germany 2018 and USUV Europe 3 the viral replication of WNV and USUV was significantly dependent on the WNV MOI and the time point post infection in all four tested cell lines. There was only one exception: the replication of USUV Europe 3 in C6/36 was not influenced by the starting MOI of WNV Germany 2018 (p=0.206). Individual differences were observed in the significance of the interaction between both factors (WNV MOI and time point) in the different cell lines for WNV and USUV (only in the GN-R was the interaction statistically significant for both USUV and WNV (p<0.0001))

**Table S12. Dependency of USUV viral replication on the virus constellation and time points in CT cells.**

| **Viral genome copies** | **Time point** | **Contrast** | **estimate** | | **SE** | | **Df** | | **t.ratio** | | **p.value** | |
| --- | --- | --- | --- | --- | --- | --- | --- | --- | --- | --- | --- | --- |
| USUV | 24 | USUV Europe 3 effect – USUV Europe 3+WNV Germany 18 0.1 effect | -0.719 | 0.0327 | | 18 | | -21.985 | | <.0001 ******* | |  |
| USUV | 24 | USUV Europe 3 effect – USUV Europe 3+WNV Germany 18 1 effect | -0.60867 | 0.0327 | | 18 | | -18.612 | | <.0001 ******* | |  |
| USUV | 24 | USUV Europe 3+WNV Germany 18 0.1 effect+ USUV Europe 3+WNV Germany 18 1 effect | 0.11033 | 0.0327 | | 18 | | 3.374 | | 0.009 ****** | |  |
| USUV | 48 | USUV Europe 3 effect – USUV Europe 3+WNV Germany 18 0.1 effect | -0.267 | 0.0327 | | 18 | | -8.164 | | <.0001 ******* | |  |
| USUV | 48 | USUV Europe 3 effect – USUV Europe 3+WNV Germany 18 1 effect | -0.12767 | 0.0327 | | 18 | | -3.904 | | 0.0028 ****** | |  |
| USUV | 48 | USUV Europe 3+WNV Germany 18 0.1 effect+ USUV Europe 3+WNV Germany 18 1 effect | 0.13933 | 0.0327 | | 18 | | 4.26 | | 0.0013 ****** | |  |
| USUV | 72 | USUV Europe 3 effect – USUV Europe 3+WNV Germany 18 0.1 effect | -0.00767 | 0.0327 | | 18 | | -0.234 | | 0.9702 | |  |
| USUV | 72 | USUV Europe 3 effect – USUV Europe 3+WNV Germany 18 1 effect | 0.42833 | 0.0327 | | 18 | | 13.097 | | <.0001 ******* | |  |
| USUV | 72 | USUV Europe 3+WNV Germany 18 0.1 effect+ USUV Europe 3+WNV Germany 18 1 effect | 0.436 | 0.0327 | | 18 | | 13.332 | | <.0001 ******* | |  |

ANOVA with PostHoc test (Least-Squares Means) showing dependency of USUV viral replication on the virus constellation and concentration of a co-infection and the time points post infection in infected CT cells (based on Figure 3). P-value adjustment was conducted using the tukey method for comparing a family of 3 estimates. Term significant at alpha = 0.05; *p<0.1, **p<0.01, ***p<0.001.

**Table S13. Dependency of WNV viral replication on the virus constellation and time points in CT cells.**

| **Viral genome copies** | **Time point** | **Viruses** | | diff | lwr | upr | p | adj |
| --- | --- | --- | --- | --- | --- | --- | --- | --- |
| WNV |  | WNV Germany 18 effect – USUV Europe 3+WNV Germany 18 0.1 effect | -0.4641111 | | -0.67598702 | -0.2522352 | 0.0000748 ******* | -0.4641111 |
| WNV |  | WNV Germany 18 effect – USUV Europe 3+WNV Germany 18 1 effect | 0.2094444 | | -0.00243147 | 0.4213204 | 0.0529829 ******* | 0.2094444 |
| WNV |  | USUV Europe 3+WNV Germany 18 0.1 effect+ USUV Europe 3+WNV Germany 18 1 effect | 0.6735556 | | 0.46167964 | 0.8854315 | 0.0000006 ******* | 0.6735556 |
| WNV | 24 effect - 48 effect |  | 1.2418889 | | 1.030013 | 1.453765 | 0 | 1.2418889 |
| WNV | 24 effect - 72 effect |  | 2.0874444 | | 1.8755685 | 2.29932 | 0 | 2.0874444 |
| WNV | 48 effect - 72 effect |  | 0.8455556 | | 0.6336796 | 1.057431 | 0 | 0.8455556 |

Tukey multiple comparisons of means test showing dependency of WNV viral replication on the virus constellation and concentration of a co-infection and the time points post infection in infected CT cells (based on Figure 3). Term significant at alpha = 0.05; *p<0.1, **p<0.01, ***p<0.001.

**Table S14. Dependence of virus combinations and different time points for C6/36 cells.**

| **Viral genome copies** | **Time point** | **Contrast** | **estimate** | **SE** | **Df** | **t.ratio** | **p.value** |
| --- | --- | --- | --- | --- | --- | --- | --- |
| WNV |  | WNV Germany 18 effect – USUV Europe 3+WNV Germany 18 0.1 effect | 0.9117 | 0.062 | 18 | 14.707 | <.0001 ******* |
| WNV |  | WNV Germany 18 effect – USUV Europe 3+WNV Germany 18 1 effect | 0.0393 | 0.062 | 18 | 0.634 | 0.8036 |
| WNV |  | USUV Europe 3+WNV Germany 18 0.1 effect+ USUV Europe 3+WNV Germany 18 1 effect | -0.8724 | 0.062 | 18 | -14.073 | <.0001 ******* |
| WNV | 24 effect - 48 effect |  | -2.01 | 0.062 | 18 | -32.404 | <.0001 ******* |
| WNV | 24 effect - 72 effect |  | -3.04 | 0.062 | 18 | -48.995 | <.0001 ******* |
| WNV | 48 effect - 72 effect |  | -1.03 | 0.062 | 18 | -16.592 | <.0001 ******* |
| USUV | 24 effect - 48 effect |  | -1.652 | 0.147 | 18 | -11.204 | <.0001 ******* |
| USUV | 24 effect - 72 effect |  | -2.27 | 0.147 | 18 | -15.397 | <.0001 ******* |
| USUV | 48 effect - 72 effect |  | -0.618 | 0.147 | 18 | -4.192 | 0.0015 ****** |
|  |  |  |  |  |  |  |  |

ANOVA with PostHoc test (Least-Squares Means) showing the dependence of virus combinations (for WNV) and different time points (for WNV and USUV) for C6/36 cells (based on Figure 3). P-value adjustment was conducted using the tukey method for comparing a family of 3 estimates. Term significant at alpha = 0.05; *p<0.1, **p<0.01, ***p<0.001.

**Table S15. Dependency of viral replication on the virus constellation and the time points in GN-R.**

| **Viral genome copies** | **Time point** | **Contrast** | **estimate** | **SE** | **Df** | **t.ratio** | **p.value** |
| --- | --- | --- | --- | --- | --- | --- | --- |
| WNV | 24 | WNV Germany 18 effect – USUV Europe 3+WNV Germany 18 0.1 effect | 1.12548 | 0.0636 | 18 | 17.707 | <.0001 ******* |
| WNV | 24 | WNV Germany 18 effect – USUV Europe 3+WNV Germany 18 1 effect | -0.01228 | 0.0636 | 18 | -0.193 | 0.9796 |
| WNV | 24 | USUV Europe 3+WNV Germany 18 0.1 effect+ USUV Europe 3+WNV Germany 18 1 effect | -1.13776 | 0.0636 | 18 | -17.9 | <.0001 ******* |
| WNV | 48 | WNV Germany 18 effect – USUV Europe 3+WNV Germany 18 0.1 effect | 0.78382 | 0.0636 | 18 | 12.331 | <.0001 ******* |
| WNV | 48 | WNV Germany 18 effect – USUV Europe 3+WNV Germany 18 1 effect | 0.71261 | 0.0636 | 18 | 11.211 | <.0001 ******* |
| WNV | 48 | USUV Europe 3+WNV Germany 18 0.1 effect+ USUV Europe 3+WNV Germany 18 1 effect | -0.07121 | 0.0636 | 18 | -1.12 | 0.5143 |
| WNV | 72 | WNV Germany 18 effect – USUV Europe 3+WNV Germany 18 0.1 effect | 0.70633 | 0.0636 | 18 | 11.112 | <.0001 ******* |
| WNV | 72 | WNV Germany 18 effect – USUV Europe 3+WNV Germany 18 1 effect | 0.71367 | 0.0636 | 18 | 11.228 | <.0001 ******* |
| WNV | 72 | USUV Europe 3+WNV Germany 18 0.1 effect+ USUV Europe 3+WNV Germany 18 1 effect | 0.00734 | 0.0636 | 18 | 0.115 | 0.9927 |
| USUV | 24 | USUV Europe 3 effect – USUV Europe 3+WNV Germany 18 1 effect | 1.637 | 0.0626 | 18 | 26.134 | <.0001 ******* |
| USUV | 24 | USUV Europe 3 effect – USUV Europe 3+WNV Germany 18 0.1 effect | 1.498 | 0.0626 | 18 | 23.921 | <.0001 ******* |
| USUV | 24 | USUV Europe 3+WNV Germany 18 0.1 effect+ USUV Europe 3+WNV Germany 18 1 effect | -0.139 | 0.0626 | 18 | -2.213 | 0.0959 ***** |
| USUV | 48 | USUV Europe 3 effect – USUV Europe 3+WNV Germany 18 1 effect | 1.903 | 0.0626 | 18 | 30.384 | <.0001 ******* |
| USUV | 48 | USUV Europe 3 effect – USUV Europe 3+WNV Germany 18 0.1 effect | 2.666 | 0.0626 | 18 | 42.566 | <.0001 ******* |
| USUV | 48 | USUV Europe 3+WNV Germany 18 0.1 effect+ USUV Europe 3+WNV Germany 18 1 effect | 0.763 | 0.0626 | 18 | 12.182 | <.0001 ******* |
| USUV | 72 | USUV Europe 3 effect – USUV Europe 3+WNV Germany 18 1 effect | 1.886 | 0.0626 | 18 | 30.105 | <.0001 ******* |
| USUV | 72 | USUV Europe 3 effect – USUV Europe 3+WNV Germany 18 0.1 effect | 2.794 | 0.0626 | 18 | 44.615 | <.0001 ******* |
| USUV | 72 | USUV Europe 3+WNV Germany 18 0.1 effect+ USUV Europe 3+WNV Germany 18 1 effect | 0.909 | 0.0626 | 18 | 14.51 | <.0001 ******* |

ANOVA with PostHoc test (Least-Squares Means) showing dependency of viral replication on the virus constellation and concentration of a co-infection and the time points post infection in infected GN-R cells (based on Figure 3). P-value adjustment was conducted using the tukey method for comparing a family of 3 estimates. Term significant at alpha = 0.05; *p<0.1, **p<0.01, ***p<0.001.

**Table S16. Dependency of viral replication on virus constellation and time point in Vero B4 and C6/36.**

| **Cell line** | **Viral genome copies** | **Factors** | **Df** | **Sum.Sq** | **Mean.Sq** | **F.value** | **Pr.(>F.)** |
| --- | --- | --- | --- | --- | --- | --- | --- |
| Vero B4 | WNV | Co-infection | 3 | 3.105 | 1.0349 | 100.89 | 9.62e-14 *** |
| Vero B4 | WNV | Time point | 2 | 3.305 | 1.6523 | 161.07 | 1.23e-14 *** |
| Vero B4 | WNV | Co-infection:Time point | 6 | 0.724 | 0.1206 | 11.76 | 3.82e-06 *** |
| Vero B4 | WNV | Residuals | 24 | 0.246 | 0.0103 |  |  |
| Vero B4 | USUV | Co-infection | 3 | 1.7401 | 0.5800 | 107.348 | 4.83e-14 *** |
| Vero B4 | USUV | Time point | 2 | 1.8507 | 0.9253 | 171.255 | 6.22e-15 *** |
| Vero B4 | USUV | Co-infection:Time point | 6 | 0.2162 | 0.0360 | 6.667 | 0.000301 *** |
| Vero B4 | USUV | Residuals | 24 | 0.1297 | 0.0054 |  |  |
| C6/36 | WNV | Co-infection | 3 | 16.29 | 5.430 | 394.195 | < 2e-16 *** |
| C6/36 | WNV | Time point | 2 | 42.61 | 21.306 | 1546.632 | < 2e-16 *** |
| C6/36 | WNV | Co-infection:Time point | 6 | 0.61 | 0.102 | 7.406 | 0.000144 *** |
| C6/36 | WNV | Residuals | 24 | 0.33 | 0.014 |  |  |
| C6/36 | USUV | Co-infection | 3 | 3.389 | 1.130 | 58.936 | 3.23e-11 *** |
| C6/36 | USUV | Time point | 2 | 20.179 | 10.090 | 526.385 | < 2e-16 *** |
| C6/36 | USUV | Co-infection:Time point | 6 | 0.417 | 0.069 | 3.624 | 0.0106 * |
| C6/36 | USUV | Residuals | 24 | 0.460 | 0.019 |  |  |

ANOVA test showing dependency of viral replication on the virus constellation of a co-infection and the Time point post infection in infected Vero B4 and C6/36 cells (based on Figure 4). Term significant at alpha = 0.05; *p<0.1, **p<0.01, ***p<0.001. To explore the relation between viral genome copies of co-infections, an ANOVA model (Viral genome copies=virus+time+virus*time) was performed using R (v3.6.2, x64). The virus replication of WNV and USUV was significantly dependent on the constellation of the co-infection as well as the time point post infection. The interaction of both factors (constellation of co-infection and time point) was also statistically significant for both cell lines (C6/36 and Vero B4). Strikingly, the viral replication of WNV Italy 2009 and Germany 2018 was statistically significant (p<0.0001 at all three time points) independent of the USUV strain with which it was co-infected (Europe 3 or Africa 3).

**Table S17. Dependency of WNV viral replication on the virus constellation and time points in Vero B4.**

| **Viral genome copies** | **Time point** | **Contrast** | **estimate** | **SE** | **Df** | **t.ratio** | **p.value** |
| --- | --- | --- | --- | --- | --- | --- | --- |
| WNV | 24 | WNV Germany 18+USUV Africa 3 effect – WNV Germany 18+USUV Europe 3 effect | -0.36492 | 0.0827 | 24 | -4.413 | 0.001 ****** |
| WNV | 24 | WNV Germany 18+USUV Africa 3 effect – WNV Italy 09+USUV Africa 3 effect | -0.56909 | 0.0827 | 24 | -6.882 | <.0001 ******* |
| WNV | 24 | WNV Germany 18+USUV Africa 3 effect – WNV Italy 09+USUV Europe 3 effect | -1.01543 | 0.0827 | 24 | -12.279 | <.0001 ******* |
| WNV | 24 | WNV Germany 18+USUV Europe 3 effect – WNV Italy 09+USUV Africa 3 effect | -0.20417 | 0.0827 | 24 | -2.469 | 0.0909 ***** |
| WNV | 24 | WNV Germany 18+USUV Europe 3 effect – WNV Italy 09+USUV Europe 3 effect | -0.6505 | 0.0827 | 24 | -7.866 | <.0001 ******* |
| WNV | 24 | WNV Italy 09+USUV Africa 3 effect – WNV Italy 09+USUV Europe 3 effect | -0.44633 | 0.0827 | 24 | -5.397 | 0.0001 ******* |
| WNV | 48 | WNV Germany 18+USUV Africa 3 effect – WNV Germany 18+USUV Europe 3 effect | 0.32831 | 0.0827 | 24 | 3.97 | 0.003 ****** |
| WNV | 48 | WNV Germany 18+USUV Africa 3 effect – WNV Italy 09+USUV Africa 3 effect | -0.00752 | 0.0827 | 24 | -0.091 | 0.9997 |
| WNV | 48 | WNV Germany 18+USUV Africa 3 effect – WNV Italy 09+USUV Europe 3 effect | -0.43019 | 0.0827 | 24 | -5.202 | 0.0001 ******* |
| WNV | 48 | WNV Germany 18+USUV Europe 3 effect – WNV Italy 09+USUV Africa 3 effect | -0.33583 | 0.0827 | 24 | -4.061 | 0.0024 ****** |
| WNV | 48 | WNV Germany 18+USUV Europe 3 effect – WNV Italy 09+USUV Europe 3 effect | -0.7585 | 0.0827 | 24 | -9.172 | <.0001 ******* |
| WNV | 48 | WNV Italy 09+USUV Africa 3 effect – WNV Italy 09+USUV Europe 3 effect | -0.42267 | 0.0827 | 24 | -5.111 | 0.0002 ******* |
| WNV | 72 | WNV Germany 18+USUV Africa 3 effect – WNV Germany 18+USUV Europe 3 effect | 0.47623 | 0.0827 | 24 | 5.759 | <.0001 ******* |
| WNV | 72 | WNV Germany 18+USUV Africa 3 effect – WNV Italy 09+USUV Africa 3 effect | -0.18979 | 0.0827 | 24 | -2.295 | 0.1274 |
| WNV | 72 | WNV Germany 18+USUV Africa 3 effect – WNV Italy 09+USUV Europe 3 effect | -0.43712 | 0.0827 | 24 | -5.286 | 0.0001 ******* |
| WNV | 72 | WNV Germany 18+USUV Europe 3 effect – WNV Italy 09+USUV Africa 3 effect | -0.66602 | 0.0827 | 24 | -8.054 | <.0001 ******* |
| WNV | 72 | WNV Germany 18+USUV Europe 3 effect – WNV Italy 09+USUV Europe 3 effect | -0.91335 | 0.0827 | 24 | -11.044 | <.0001 ******* |
| WNV | 72 | WNV Italy 09+USUV Africa 3 effect – WNV Italy 09+USUV Europe 3 effect | -0.24733 | 0.0827 | 24 | -2.991 | 0.0301 ***** |

ANOVA with PostHoc test (Least-Squares Means) showing dependency of WNV viral replication on the virus constellation of a co-infection and the time points post infection in infected Vero B4 cells (based on Figure 4). P-value adjustment was conducted using the tukey method for comparing a family of 3 estimates. Term significant at alpha = 0.05; *p<0.1, **p<0.01, ***p<0.001.

**Table S18. Dependency of USUV viral replication on the virus constellation and time points in Vero B4.**

| **Viral genome copies** | **Time point** | **Contrast** | **estimate** | **SE** | **Df** | **t.ratio** | **p.value** |
| --- | --- | --- | --- | --- | --- | --- | --- |
| USUV | 24 | WNV Germany 18+USUV Africa 3 effect – WNV Germany 18+USUV Europe 3 effect | -0.7551 | 0.06 | 24 | -12.581 | <.0001 ******* |
| USUV | 24 | WNV Germany 18+USUV Africa 3 effect – WNV Italy 09+USUV Africa 3 effect | -0.4995 | 0.06 | 24 | -8.323 | <.0001 ******* |
| USUV | 24 | WNV Germany 18+USUV Africa 3 effect – WNV Italy 09+USUV Europe 3 effect | -0.7137 | 0.06 | 24 | -11.892 | <.0001 ******* |
| USUV | 24 | WNV Germany 18+USUV Europe 3 effect – WNV Italy 09+USUV Africa 3 effect | 0.2555 | 0.06 | 24 | 4.258 | 0.0015 ****** |
| USUV | 24 | WNV Germany 18+USUV Europe 3 effect – WNV Italy 09+USUV Europe 3 effect | 0.0413 | 0.06 | 24 | 0.689 | 0.9004 |
| USUV | 24 | WNV Italy 09+USUV Africa 3 effect – WNV Italy 09+USUV Europe 3 effect | -0.2142 | 0.06 | 24 | -3.569 | 0.0079 ****** |
| USUV | 48 | WNV Germany 18+USUV Africa 3 effect – WNV Germany 18+USUV Europe 3 effect | -0.3378 | 0.06 | 24 | -5.627 | <.0001 ******* |
| USUV | 48 | WNV Germany 18+USUV Africa 3 effect – WNV Italy 09+USUV Africa 3 effect | -0.0298 | 0.06 | 24 | -0.497 | 0.959 |
| USUV | 48 | WNV Germany 18+USUV Africa 3 effect – WNV Italy 09+USUV Europe 3 effect | -0.4094 | 0.06 | 24 | -6.822 | <.0001 ******* |
| USUV | 48 | WNV Germany 18+USUV Europe 3 effect – WNV Italy 09+USUV Africa 3 effect | 0.3079 | 0.06 | 24 | 5.13 | 0.0002 ******* |
| USUV | 48 | WNV Germany 18+USUV Europe 3 effect – WNV Italy 09+USUV Europe 3 effect | -0.0717 | 0.06 | 24 | -1.194 | 0.6364 |
| USUV | 48 | WNV Italy 09+USUV Africa 3 effect – WNV Italy 09+USUV Europe 3 effect | -0.3796 | 0.06 | 24 | -6.325 | <.0001 ******* |
| USUV | 72 | WNV Germany 18+USUV Africa 3 effect – WNV Germany 18+USUV Europe 3 effect | -0.425 | 0.06 | 24 | -7.081 | <.0001 ******* |
| USUV | 72 | WNV Germany 18+USUV Africa 3 effect – WNV Italy 09+USUV Africa 3 effect | -0.246 | 0.06 | 24 | -4.098 | 0.0022 ****** |
| USUV | 72 | WNV Germany 18+USUV Africa 3 effect – WNV Italy 09+USUV Europe 3 effect | -0.5267 | 0.06 | 24 | -8.775 | <.0001 ******* |
| USUV | 72 | WNV Germany 18+USUV Europe 3 effect – WNV Italy 09+USUV Africa 3 effect | 0.179 | 0.06 | 24 | 2.983 | 0.0307 ***** |
| USUV | 72 | WNV Germany 18+USUV Europe 3 effect – WNV Italy 09+USUV Europe 3 effect | -0.1017 | 0.06 | 24 | -1.694 | 0.3486 |
| USUV | 72 | WNV Italy 09+USUV Africa 3 effect – WNV Italy 09+USUV Europe 3 effect | -0.2807 | 0.06 | 24 | -4.677 | 0.0005 ******* |

ANOVA with PostHoc test (Least-Squares Means) showing dependency of USUV viral replication on the virus constellation of a co-infection and the time points post infection in infected Vero B4 cells (based on Figure 4). P-value adjustment was conducted using the tukey method for comparing a family of 3 estimates. Term significant at alpha = 0.05; *p<0.1, **p<0.01, ***p<0.001.

**Table S19. Dependency of WNV viral replication on the virus constellation and time points in C6/36.**

| **Viral genome copies** | **Time point** | **Contrast** | **estimate** | **SE** | **Df** | **t.ratio** | **p.value** |
| --- | --- | --- | --- | --- | --- | --- | --- |
| WNV | 24 | WNV Germany 18+USUV Africa 3 effect – WNV Germany 18+USUV Europe 3 effect | -0.0977 | 0.0958 | 24 | -1.019 | 0.7399 |
| WNV | 24 | WNV Germany 18+USUV Africa 3 effect – WNV Italy 09+USUV Africa 3 effect | -1.4167 | 0.0958 | 24 | -14.783 | <.0001 ******* |
| WNV | 24 | WNV Germany 18+USUV Africa 3 effect – WNV Italy 09+USUV Europe 3 effect | -1.7867 | 0.0958 | 24 | -18.644 | <.0001 ******* |
| WNV | 24 | WNV Germany 18+USUV Europe 3 effect – WNV Italy 09+USUV Africa 3 effect | -1.319 | 0.0958 | 24 | -13.764 | <.0001 ******* |
| WNV | 24 | WNV Germany 18+USUV Europe 3 effect – WNV Italy 09+USUV Europe 3 effect | -1.689 | 0.0958 | 24 | -17.625 | <.0001 ******* |
| WNV | 24 | WNV Italy 09+USUV Africa 3 effect – WNV Italy 09+USUV Europe 3 effect | -0.37 | 0.0958 | 24 | -3.861 | 0.0039 ****** |
| WNV | 48 | WNV Germany 18+USUV Africa 3 effect – WNV Germany 18+USUV Europe 3 effect | -0.1397 | 0.0958 | 24 | -1.458 | 0.4772 |
| WNV | 48 | WNV Germany 18+USUV Africa 3 effect – WNV Italy 09+USUV Africa 3 effect | -1.3237 | 0.0958 | 24 | -13.813 | <.0001 ******* |
| WNV | 48 | WNV Germany 18+USUV Africa 3 effect – WNV Italy 09+USUV Europe 3 effect | -1.6684 | 0.0958 | 24 | -17.409 | <.0001 ******* |
| WNV | 48 | WNV Germany 18+USUV Europe 3 effect – WNV Italy 09+USUV Africa 3 effect | -1.1839 | 0.0958 | 24 | -12.355 | <.0001 ******* |
| WNV | 48 | WNV Germany 18+USUV Europe 3 effect – WNV Italy 09+USUV Europe 3 effect | -1.5286 | 0.0958 | 24 | -15.951 | <.0001 ******* |
| WNV | 48 | WNV Italy 09+USUV Africa 3 effect – WNV Italy 09+USUV Europe 3 effect | -0.3447 | 0.0958 | 24 | -3.597 | 0.0074 ****** |
| WNV | 72 | WNV Germany 18+USUV Africa 3 effect – WNV Germany 18+USUV Europe 3 effect | -0.3446 | 0.0958 | 24 | -3.596 | 0.0074 ****** |
| WNV | 72 | WNV Germany 18+USUV Africa 3 effect – WNV Italy 09+USUV Africa 3 effect | -1.0395 | 0.0958 | 24 | -10.847 | <.0001 ******* |
| WNV | 72 | WNV Germany 18+USUV Africa 3 effect – WNV Italy 09+USUV Europe 3 effect | -1.2662 | 0.0958 | 24 | -13.212 | <.0001 ******* |
| WNV | 72 | WNV Germany 18+USUV Europe 3 effect – WNV Italy 09+USUV Africa 3 effect | -0.6949 | 0.0958 | 24 | -7.251 | <.0001 ******* |
| WNV | 72 | WNV Germany 18+USUV Europe 3 effect – WNV Italy 09+USUV Europe 3 effect | -0.9216 | 0.0958 | 24 | -9.617 | <.0001 ******* |
| WNV | 72 | WNV Italy 09+USUV Africa 3 effect – WNV Italy 09+USUV Europe 3 effect | -0.2267 | 0.0958 | 24 | -2.365 | 0.1114 |

ANOVA with PostHoc test (Least-Squares Means) showing dependency of WNV viral replication on the virus constellation of a co-infection and the time points post infection in infected C6/36 cells (based on Figure 4). P-value adjustment was conducted using the tukey method for comparing a family of 3 estimates. Term significant at alpha = 0.05; *p<0.1, **p<0.01, ***p<0.001.

**Table S20. Dependency of USUV viral replication on the virus constellation and time points in C6/36.**

| **Viral genome copies** | **Time point** | **Contrast** | **estimate** | **SE** | **Df** | **t.ratio** | **p.value** |
| --- | --- | --- | --- | --- | --- | --- | --- |
| USUV | 24 | WNV Germany 18+USUV Africa 3 effect – WNV Germany 18+USUV Europe 3 effect | -0.7724 | 0.113 | 24 | -6.833 | <.0001 ******* |
| USUV | 24 | WNV Germany 18+USUV Africa 3 effect – WNV Italy 09+USUV Africa 3 effect | -0.3274 | 0.113 | 24 | -2.897 | 0.0371 ***** |
| USUV | 24 | WNV Germany 18+USUV Africa 3 effect – WNV Italy 09+USUV Europe 3 effect | -1.1318 | 0.113 | 24 | -10.012 | <.0001 ******* |
| USUV | 24 | WNV Germany 18+USUV Europe 3 effect – WNV Italy 09+USUV Africa 3 effect | 0.445 | 0.113 | 24 | 3.937 | 0.0032 ****** |
| USUV | 24 | WNV Germany 18+USUV Europe 3 effect – WNV Italy 09+USUV Europe 3 effect | -0.3593 | 0.113 | 24 | -3.179 | 0.0197 ***** |
| USUV | 24 | WNV Italy 09+USUV Africa 3 effect – WNV Italy 09+USUV Europe 3 effect | -0.8043 | 0.113 | 24 | -7.115 | <.0001 ******* |
| USUV | 48 | WNV Germany 18+USUV Africa 3 effect – WNV Germany 18+USUV Europe 3 effect | -0.534 | 0.113 | 24 | -4.724 | 0.0005 ******* |
| USUV | 48 | WNV Germany 18+USUV Africa 3 effect – WNV Italy 09+USUV Africa 3 effect | -0.3815 | 0.113 | 24 | -3.375 | 0.0125 ***** |
| USUV | 48 | WNV Germany 18+USUV Africa 3 effect – WNV Italy 09+USUV Europe 3 effect | -0.8357 | 0.113 | 24 | -7.393 | <.0001 ******* |
| USUV | 48 | WNV Germany 18+USUV Europe 3 effect – WNV Italy 09+USUV Africa 3 effect | 0.1525 | 0.113 | 24 | 1.349 | 0.5421 |
| USUV | 48 | WNV Germany 18+USUV Europe 3 effect – WNV Italy 09+USUV Europe 3 effect | -0.3017 | 0.113 | 24 | -2.669 | 0.0605 ***** |
| USUV | 48 | WNV Italy 09+USUV Africa 3 effect – WNV Italy 09+USUV Europe 3 effect | -0.4542 | 0.113 | 24 | -4.018 | 0.0027 ****** |
| USUV | 72 | WNV Germany 18+USUV Africa 3 effect – WNV Germany 18+USUV Europe 3 effect | -0.3391 | 0.113 | 24 | -3 | 0.0295 ***** |
| USUV | 72 | WNV Germany 18+USUV Africa 3 effect – WNV Italy 09+USUV Africa 3 effect | -0.3629 | 0.113 | 24 | -3.21 | 0.0183 ***** |
| USUV | 72 | WNV Germany 18+USUV Africa 3 effect – WNV Italy 09+USUV Europe 3 effect | -0.5688 | 0.113 | 24 | -5.032 | 0.0002 ******* |
| USUV | 72 | WNV Germany 18+USUV Europe 3 effect – WNV Italy 09+USUV Africa 3 effect | -0.0238 | 0.113 | 24 | -0.21 | 0.9966 |
| USUV | 72 | WNV Germany 18+USUV Europe 3 effect – WNV Italy 09+USUV Europe 3 effect | -0.2297 | 0.113 | 24 | -2.032 | 0.2046 |
| USUV | 72 | WNV Italy 09+USUV Africa 3 effect – WNV Italy 09+USUV Europe 3 effect | -0.2059 | 0.113 | 24 | -1.821 | 0.2882 |

ANOVA with PostHoc test (Least-Squares Means) showing dependency of USUV viral replication on the virus constellation of a co-infection and the time points post infection in infected C6/36 cells (based on Figure 4). P-value adjustment was conducted using the tukey method for comparing a family of 3 estimates. Term significant at alpha = 0.05; *p<0.1, **p<0.01, ***p<0.001.
